# PERK-mediated expression of peptidylglycine α-amidating monooxygenase supports angiogenesis in glioblastoma

**DOI:** 10.1038/s41389-020-0201-8

**Published:** 2020-02-13

**Authors:** Himanshu Soni, Julia Bode, Chi D. L. Nguyen, Laura Puccio, Michelle Neßling, Rosario M. Piro, Jonas Bub, Emma Phillips, Robert Ahrends, Betty A. Eipper, Björn Tews, Violaine Goidts

**Affiliations:** 1 Brain Tumor Translational Targets, DKFZ Junior Group, German Cancer Research Center (DKFZ), Heidelberg, Germany; 2 Molecular Mechanisms of Tumor Invasion, Schaller Research Group, University of Heidelberg and German Cancer Research Center (DKFZ), Heidelberg, Germany; 3 Leibniz-Institut für Analytische Wissenschaften—ISAS—e.V, Dortmund, Germany; 4 Central Unit Electron Microscopy, German Cancer Research Center (DKFZ), Heidelberg, Germany; 5 Institute of Computer Science, Institute of Bioinformatics, Freie Universität Berlin, Berlin, Germany; 6 Institute of Medical Genetics and Human Genetics, Charité-Universitätsmedizin Berlin, Berlin, Germany; 7 German Cancer Consortium (DKTK) partner site Berlin, and German Cancer Research Center (DKFZ), Heidelberg, Germany; 8 Department of Analytical Chemistry, Faculty of Chemistry, University of Vienna, Wien, Austria; 9 UConn Health, Farmington, CT USA

**Keywords:** CNS cancer, Tumour angiogenesis

## Abstract

PKR-like kinase (PERK) plays a significant role in inducing angiogenesis in various cancer types including glioblastoma. By proteomics analysis of the conditioned medium from a glioblastoma cell line treated with a PERK inhibitor, we showed that peptidylglycine α-amidating monooxygenase (PAM) expression is regulated by PERK under hypoxic conditions. Moreover, PERK activation via CCT020312 (a PERK selective activator) increased the cleavage and thus the generation of PAM cleaved cytosolic domain (PAM sfCD) that acts as a signaling molecule from the cytoplasm to the nuclei. PERK was also found to interact with PAM, suggesting a possible involvement in the generation of PAM sfCD. Knockdown of *PERK* or *PAM* reduced the formation of tubes by HUVECs in vitro. Furthermore, in vivo data highlighted the importance of PAM in the growth of glioblastoma with reduction of PAM expression in engrafted tumor significantly increasing the survival in mice. In summary, our data revealed PAM as a potential target for antiangiogenic therapy in glioblastoma.

## Introduction

Glioblastoma is a highly aggressive primary brain tumor with less than 15 months of median patient survival^1^. Reasons behind this poor prognosis include a high rate of angiogenesis, diffuse growth and modulation of the tumor microenvironment. PKR-like kinase (PERK) has been shown to play a significant role in enhancing the level of angiogenesis within different tumor entities including glioblastoma by regulating VEGF expression^2,3^.

PERK is one of the three unfolded protein response (UPR) sensors that become activated under endoplasmic reticulum stress conditions and functions to reduce the accumulation of misfolded proteins within the ER. It oligomerizes upon detection of misfolded proteins and is activated by auto-phosphorylation at threonine 980 on its cytosolic domain. This type-I membrane protein kinase inhibits the translation machinery of a cell by phosphorylating eIF2α at serine 51 among other known targets^4,5^. PERK has also been found to be involved in the secretion of collagen, insulin and generation of damage-associated molecular patterns (DAMPs)^6,7^.

The antiangiogenic therapies used until now, which mostly target the VEGF signaling pathway and include Bevacizumab (targeting VEGFa), have shown no benefit to overall survival for glioblastoma patients^8^. This could be explained by several resistance mechanisms, including the activation of angiogenesis via alternative proangiogenic factors^9,10^. Therefore, there is an urgent need to find new potential angiogenic targets in glioblastoma. Here, we provide evidence of PERK-mediated regulation of angiogenesis via peptidylglycine α-amidating monooxygenase (PAM). Our data support the role of PAM in tumor progression introducing the molecule as a therapeutic target against glioblastoma.

## Results

### PERK-mediated secretion of proteins under hypoxia

**Fig. 1:**
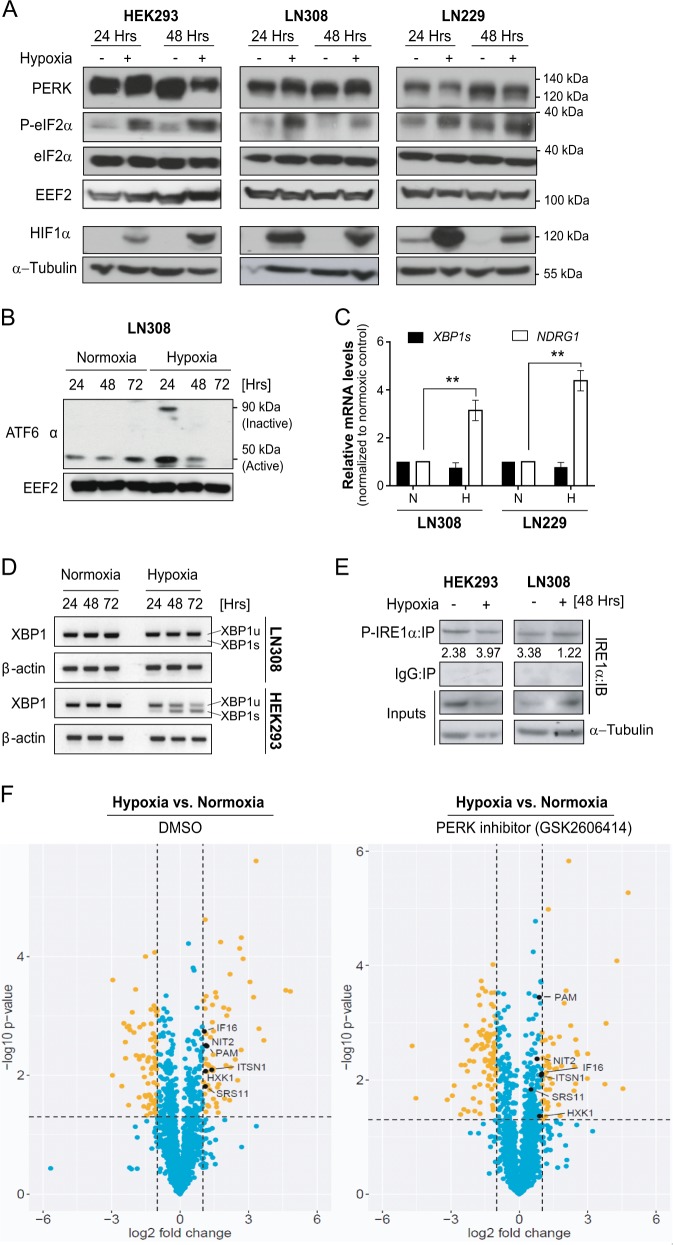
PERK-mediated secretion of proteins under hypoxia. **a** Total PERK, eIF2α and P-eIF2α expression in HEK293, LN308 and LN229 cell lines under normoxia or hypoxia. **b** Active form of ATF6α (50 kDa band) in LN308, under hypoxia for 24, 48 and 72 h. EEF2 was used as a loading control. **c** Relative mRNA levels of *XBP1s* as determined by qRT-PCR in LN308 and LN229 cell lines under hypoxia (48 h). *NDRG1* was taken as a positive control for hypoxia induction. *EEF2* was used as housekeeping gene. Data are normalized to the respective normoxic conditions and are represented as the mean of three independent experiments ± SEM (*T* test: **p value < 0.01). N normoxia, H hypoxia. **d** Total *XBP1s* mRNA transcripts in HEK293 and LN308 cells treated with hypoxia for 24, 48, 72 h. β-actin was used as a housekeeping gene. **e** Total P-IRE1α species immunoprecipitated using P-IRE1α antibody from HEK293 and LN308 cells treated with hypoxia for 48 h. **f** Volcano plot representing the regulated secretory proteins from LN308 glioblastoma cells under hypoxic conditions for 72 h without (left) and with PERK inhibitor (GSK2606414; right). The data are represented as the mean of three independent replicates. The significant *p* value cut-off was set at 0.05.

In order to determine which UPR branch is active in glioblastoma under hypoxic conditions, LN308 and LN229 glioblastoma cells were treated with 1% O_2_ and the UPR pathway was analyzed. Activation of PERK (as indicated by mobility shift in total PERK and phosphorylation of eIF2α) was evident in the control and glioblastoma cells under hypoxia (Fig. 1a). Hypoxia also resulted in the formation of active cleaved ATF6α (50 kDa) initially, but this decreased after 48 and 72 h of hypoxia treatment indicating a potential adaptation to the stress conditions (Fig. 1b). Unlike in HEK293 cells, IRE1α was not active in glioblastoma under hypoxia as demonstrated by the absence of *XBP1s* mRNA (a downstream product of the active-IRE1α RNase domain) and by the decrease in phosphorylated IRE1α (necessary for the activation of its kinase activity) (Fig. 1c–e). The data suggest that LN308 cells are better equipped to tolerate hypoxia than HEK293 cells and highlight a possible selective activation of UPR branches under hypoxia in glioblastoma in vitro.

In order to identify secretory proteins regulated by PERK in glioblastoma cells under hypoxia, LN308 cells were cultivated and treated with GSK2606414, a PERK inhibitor, under normoxic or hypoxic conditions for 72 h (Supplementary Fig. S1A). Proteomics analysis of the conditioned media was performed to identify secreted proteins that are regulated by PERK under hypoxia (Fig. 1f). Among the identified hits (Table 1), PAM was the only known protein to have its luminal domains secreted outside of the cell and is thereby a potential angiogenic candidate regulated by PERK in glioblastoma.

**Table 1:** List of proteins found to be significantly regulated by PERK under hypoxia.

Accession number	Description	DMSO^a^	GSK2606414^a^
P19021	Peptidyl-glycine alpha-amidating monooxygenase (PAM)	2.26	1.83
P19367	Hexokinase-1 (HXK1)	2.17	1.85
Q05519	Serine/arginine-rich splicing factor 11 (SRS11)	2.16	1.43
Q15811	Intersectin-1 (ITSN1)	2.61	1.95
Q16666	Gamma-interferon-inducible protein 16 (IF16)	2.08	1.97
Q9NQR4	Omega-amidase NIT2 (NIT2)	2.19	1.71

^a^Fold-change protein expression level normalized to the respective normoxic conditions, as determined by mass spectrometry analysis.

### PERK regulates PAM at mRNA level independent of PERK kinase activity

**Fig. 2:**
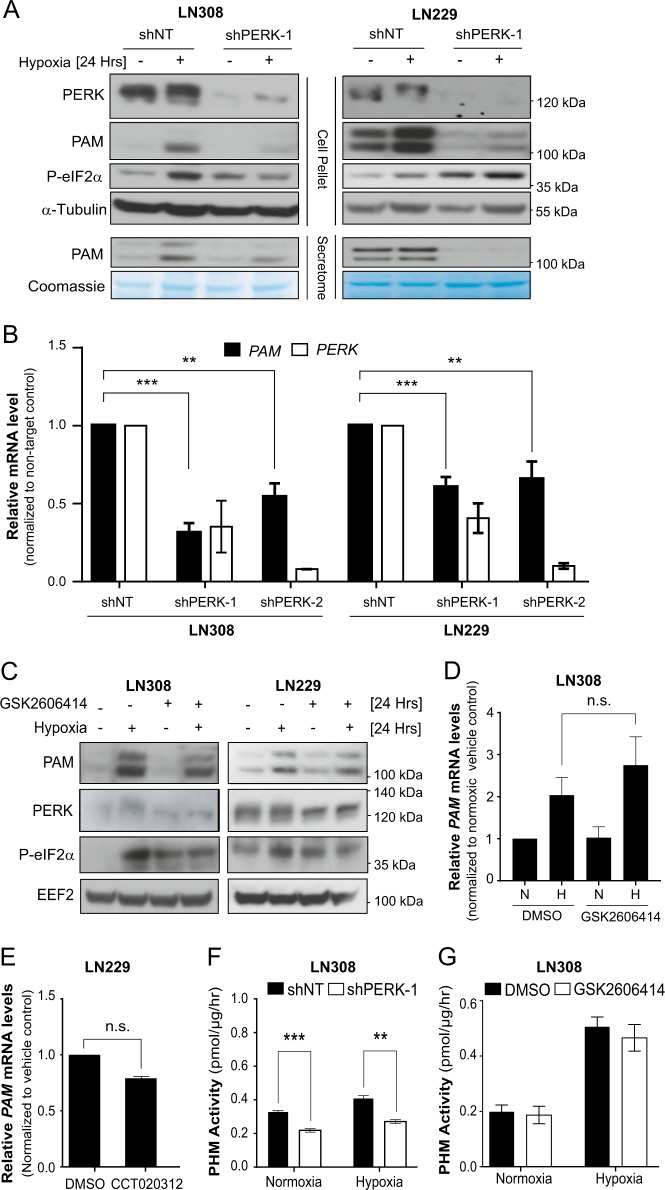
PERK regulates PAM at mRNA level independent of PERK kinase activity. **a** PAM precipitated from conditioned media of LN308 and LN229 glioblastoma cells expressing either shNT or shPERK-1 (using 10% TCA protocol). Equal amounts of protein was loaded from the harvested conditioned media. Coomassie staining and α-tubulin were used as loading controls for conditioned media (secretome) and cells, respectively. Cells were cultured in serum-free conditions. **b** Relative mRNA levels of *PAM* in LN308 and LN229 cells after cultivation under 24 h of hypoxia with PERK silencing using shPERK-1 and shPERK-2. Data were normalized to housekeeper *EEF2* and are represented as the mean of three independent replicates ± SEM; *t* test with *p* value < 0.01** and <0.001***. **c** Levels of PAM protein under PERK inhibition using GSK2606414 (500 nM) in LN308 and LN229 cells under hypoxia. **d** Relative *PAM* mRNA levels in LN308 cells under PERK inhibition using GSK2606414 (500 nM) when cultivated under hypoxia for 24 h (mean of three independent replicates ± SEM; n.s. not significant). N normoxia, H hypoxia. **e** Relative *PAM* mRNA level from LN229 cells treated with 4 μM of CCT020312 under hypoxia (24 h). Data were normalized to the housekeeper *RPS13* and are represented as the mean of three independent experiments ± SEM (*t* test with n.s. not significant). PHM activity was quantified from LN308 cells with either **f** PERK silencing or **g** PERK inhibition under hypoxia for 24 h. The data are represented as the mean of three independent experiments ± SEM; *t* test with *p* value < 0.01** and <0.001***.

In order to verify the involvement of PERK in the regulation of PAM in glioblastoma and thereby validating the mass spectrometry data, we silenced PERK in LN308 and LN229 cells under hypoxia and determined the levels of PAM in the conditioned media. PAM protein secretion increased under hypoxia and strongly decreased upon silencing of PERK (Fig. 2a). Next, we investigated whether PERK kinase activity is necessary for regulating PAM expression. While PERK knockdown caused a significant decrease in PAM protein and mRNA levels in LN308 cells and in LN229 cells (Fig. 2a, b and Supplementary Fig. S2A), its kinase inhibition or activation showed a minimal effect on the PAM protein levels and did not show any effect on *PAM* mRNA levels (Fig. 2c, e), suggesting that the kinase activity of PERK is not the major regulator of PAM. In order to determine whether PERK also affects the activity of PAM, we measured the hydroxylating activity of PAM hydroxylating monooxygenase domain (PHM), which decreased upon *PERK* knockdown (Fig. 2f), but did not change when PERK kinase activity was inhibited (Fig. 2g and Supplementary Fig. S2B), indicating that the decrease in hydroxylating activity observed upon PERK knockdown was due to decreased PAM levels. The results were confirmed in a low-passage patient-derived glioblastoma primary cell line (NCH82; Supplementary Fig. S2C−E). We conclude that PERK is essential for the expression of *PAM* mRNA, but that this is independent of the kinase activity of PERK.

### PERK activation causes accumulation of PAM sfCD fragment, possibly via a physical interaction

**Fig. 3:**
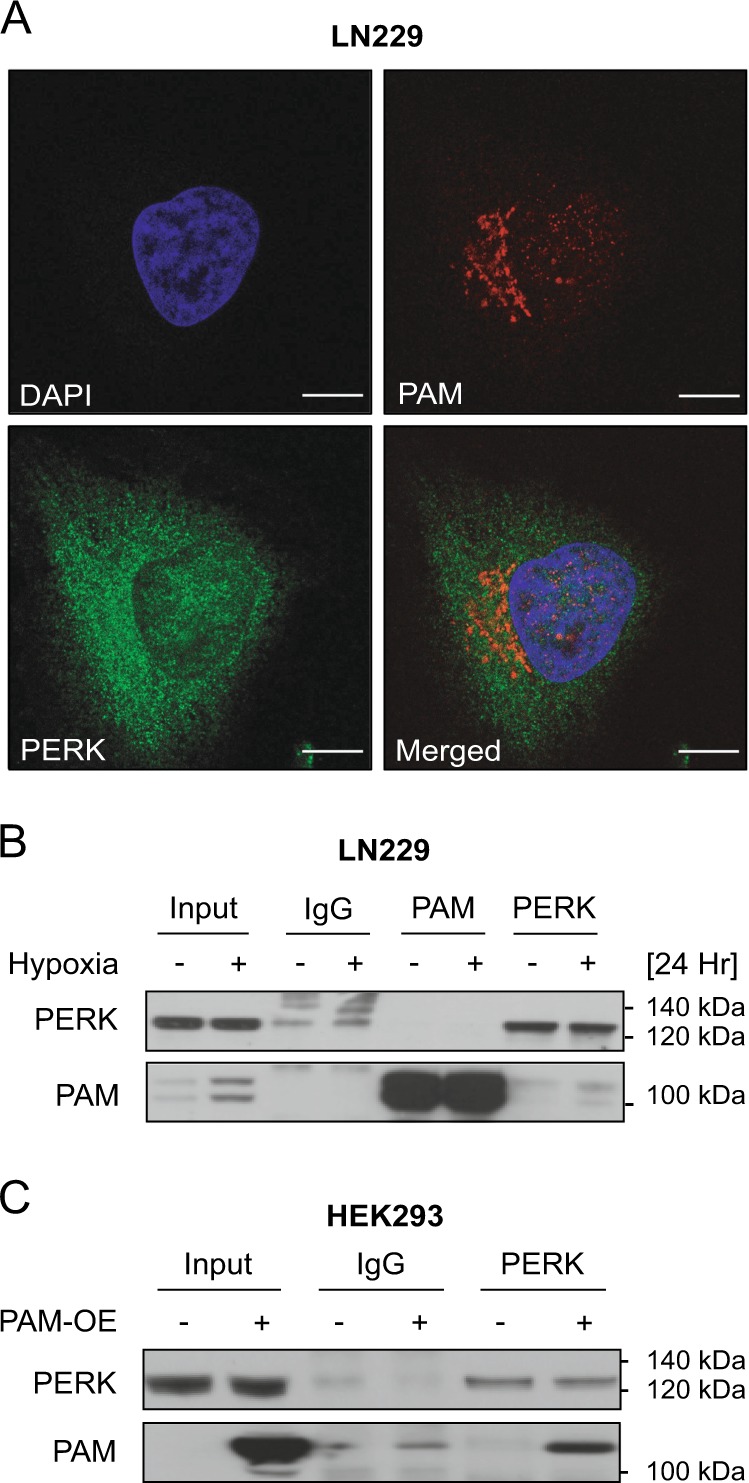
PERK interacts with PAM protein. **a** PAM and PERK immunofluorescence images from LN229 cells treated with hypoxia for 24 h. DAPI was used as a nuclear stain. Scale bars: 10 μm. **b** Levels of PAM and PERK in co-immunoprecipitation experiments using respective antibodies in LN229 cells. α-PAM or α-PERK antibodies were used to precipitate PAM or PERK respectively, from LN229 protein lysates. **c** PAM co-IP with PERK antibody from HEK293 cells overexpressing PAM protein.

**Fig. 4:**
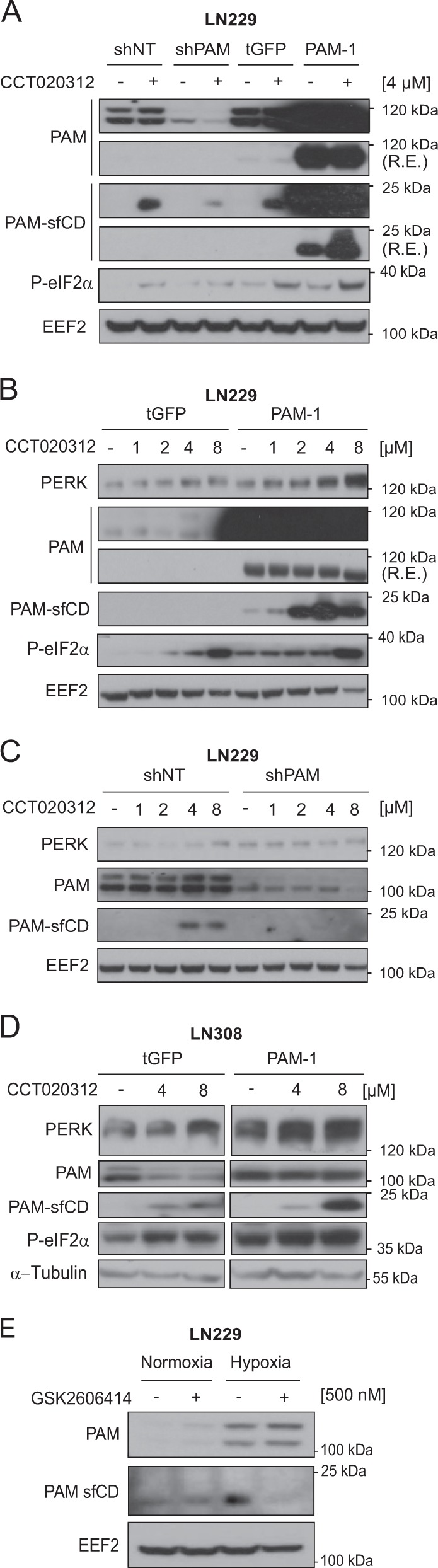
PERK activation causes accumulation of the PAM sfCD fragment. **a** sfCD PAM levels in LN229 cells with *PAM* knockdown or with PAM overexpression treated with 4 μM CCT020312 under hypoxia for 24 h. Plasmid-overexpressing turboGFP (tGFP) was used as negative control. sfCD PAM levels in LN229 cells with either **b** PAM overexpression or **c** knockdown were treated with different concentrations of CCT020312 for 24 h under normoxia. Plasmid-overexpressing turboGFP (tGFP) was used as negative control. **d** PAM sfCD levels in LN308 cells overexpressing either tGFP or PAM, treated with different concentrations of CCT020312 for 24 h. α-tubulin was used as a loading control. **e** PAM sfCD levels in LN229 cells treated with 500 nM GSK2606414 for 24 h under hypoxia. R.E. reduced exposure.

Both PAM and PERK are type-I membrane proteins residing on the ER and Golgi membranes respectively (Supplementary Fig. S3A and B). Using immunofluorescence staining and confocal microscopy, we observed that PERK and PAM are in close proximity to each other in LN229 in hypoxia and normoxia (Fig. 3a and Supplementary Fig. S3C), which prompted us to investigate whether they interact with each other. For this, we immunoprecipitated PERK and PAM from LN229 cells kept under hypoxia for 24 h, and from HEK293 overexpressing PAM. In both the cases we observed an interaction between PERK and PAM (Fig. 3b, c). As PERK kinase activity does not seem to be a major regulator of PAM at the mRNA level, we asked whether active PERK is involved in the posttranslational modification of PAM, using CCT020312, a PERK-specific activator that does not activate the IRE1α and ATF6α branches of the UPR. PAM is composed of several domains, including the unstructured cytosolic domain that can be cleaved to generate sfCD (soluble fragment-cytosolic domain). PAM sfCD has been previously shown to localize to the nucleus and suggested to be involved in the regulation of gene expression. LN229 cells with PAM overexpression or knockdown were treated with CCT020312 under hypoxia (Fig. 4a) and normoxia (Fig. 4b, c). Interestingly, we observed an increased amount of the 16 kDa PAM sfCD, a phenomenon that occurs in a concentration-dependent manner and happens independently of the oxygen level. PERK activation with CCT020312 also increased PAM sfCD in LN308 glioblastoma cells with and without PAM overexpression under hypoxia (Fig. 4d). Interestingly, CCT020312 treatment in LN308 cells (overexpressing tGFP as a negative control) reduced the total levels of the full-length PAM protein, which could be a result of subsequent degradation or secretion of the PAM luminal domain.

To determine the cellular localization of PAM sfCD in glioblastoma, we treated LN308 cells with either PERK inhibitor GSK2606414 or PERK activator CCT020312 under hypoxia and normoxia control and performed nuclear and cytoplasmic fractionation. Interestingly, we observed an increased nuclear localization of PAM sfCD under hypoxia, which increased upon strong activation of PERK by CCT020312 treatment (Supplementary Fig. S3D). Furthermore, PERK inhibitor GSK2606414 was able to reduce the nuclear localization of PAM sfCD in LN308 cells. Treatment of LN229 cells with GSK reduced total levels of PAM sfCD (Fig. 4e) without inducing changes at mRNA level (Fig. 2d, e). Our data highlight the importance of PERK kinase activity in regulating the generation of PAM sfCD.

### PAM mRNA increase under hypoxia is HIF1α-dependent

**Fig. 5:**
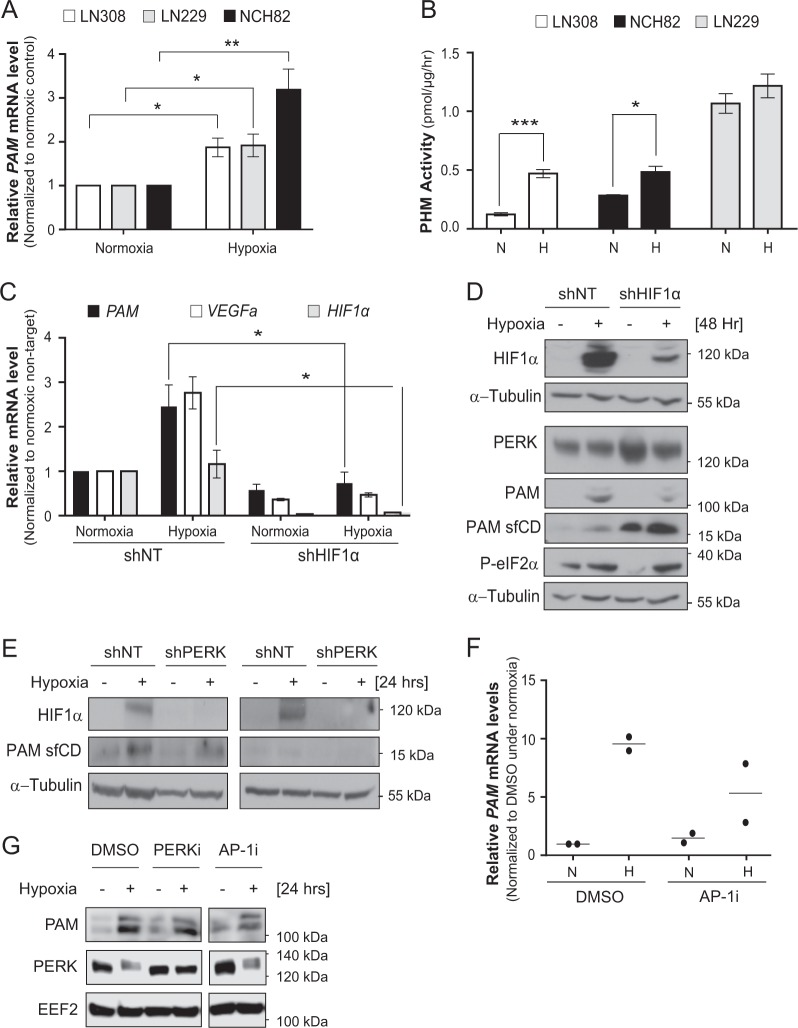
*PAM* mRNA increase under hypoxia is HIF1α dependent. **a** Levels of *PAM* mRNA in different glioblastoma cell lines under hypoxia for 24 h. Data are normalized to housekeeping gene *EEF2* and the respective normoxic conditions, and are represented as the mean of three independent experiments ± SEM (*t* test: *p* value < 0.05* and <0.01**). **b** PHM activity in different glioblastoma cell lines under 24 h of hypoxia. The experiments were performed in three independent biological replicates ± SEM (*t* test: *p* value < 0.05* and <0.001***). N normoxia, H hypoxia. **c** Relative mRNA (mean of three independent replicates ± SEM; *t* test with *p* value < 0.05*) and protein **d** levels of PAM and PAM sfCD under nontarget or *HIF1α* knockdown in LN308 cells. *EEF2* was used as housekeeping gene for RT-PCR in (**c**). **e** HIF1α and PAM sfCD protein levels in glioblastoma cells upon PERK silencing when cultivated under hypoxia for 24 h. EEF2 was used as a loading control. The same protein extracts were also used in Fig. 2a. **f** Relative *PAM* mRNA level in the presence of AP-1i (SR11302) in LN308 cells under 24 h of hypoxia. The data are represented as a mean of two independent biological replicates. RPS13 was used as housekeeper gene. **g** PAM protein level under hypoxia with PERK kinase inhibition (PERKi; GSK2606414 (500 nM)) and AP-1i (SR11302; 2 µM). EEF2 was used as a loading control.

Hypoxia regulates the expression of PAM at mRNA (Fig. 5a) and protein levels (Supplementary Fig. S4A and B). CoCl_2_, a potent inducer of hypoxia, also increases PAM protein levels in LN308 cells (Supplementary Fig. S4C). In addition, hypoxia leads to an increase in PHM activity of glioblastoma cells (Fig. 5b). In order to determine whether hypoxia inducible factor 1α (HIF1α) is responsible for the increased PAM expression under hypoxia, LN308 cells were transduced with shNT or shHIF1α and cultivated under hypoxia. *HIF1α* knockdown significantly reduced the level of PAM at the mRNA and protein level (Fig. 5c, d). Moreover, PERK silencing led to reduced HIF1α protein levels (Fig. 5e). This suggests that the effect of PERK on *PAM* mRNA under hypoxia is mediated by HIF1α. Notably, PAM sfCD levels increased upon HIF1α knockdown in LN308 cells but did not seem to be affected upon PERK knockdown (Fig. 5d, e). This suggests that the effect of PERK on the cleavage of PAM is not dependent on HIF1α. Taken together, the data suggest that the regulation of PAM expression and cleavage are regulated by PERK through discrete mechanisms.

As PERK and HIF1α both regulate PAM at the mRNA level, we took an *in silico* approach in order to identify potential transcription factors (TFs) of PAM. We performed comparative analysis of transcription binding affinities (TBAs) of all TFs and generated a list with TFs having the highest TBAs for the PAM promoter (NM_138821) defined as −1500 bases to +500 base pairs from the transcription start site (Supplementary Fig. S4D and Supplementary Table S1). From the putative transcription factors identified, several members of the AP-1 transcription complex such as FOSL1, JUN and JUNB were among the top hits (Supplementary Fig. S4E and F). HIF1α was not found listed among the top hits in this *in silico* analysis. To determine whether AP-1 is involved in the regulation of *PAM* mRNA in glioblastoma, we inhibited AP-1 in LN308 cells using the AP-1 inhibitor SR11302 (AP-1i) under hypoxia. The AP-1i reduced PAM mRNA and protein levels (Fig. 5f, g), suggesting that the AP-1 complex is a relevant factor for the regulation of PAM expression in glioblastoma.

### PAM expression is necessary for angiogenesis in vitro and progression of glioblastoma in vivo

**Fig. 6:**
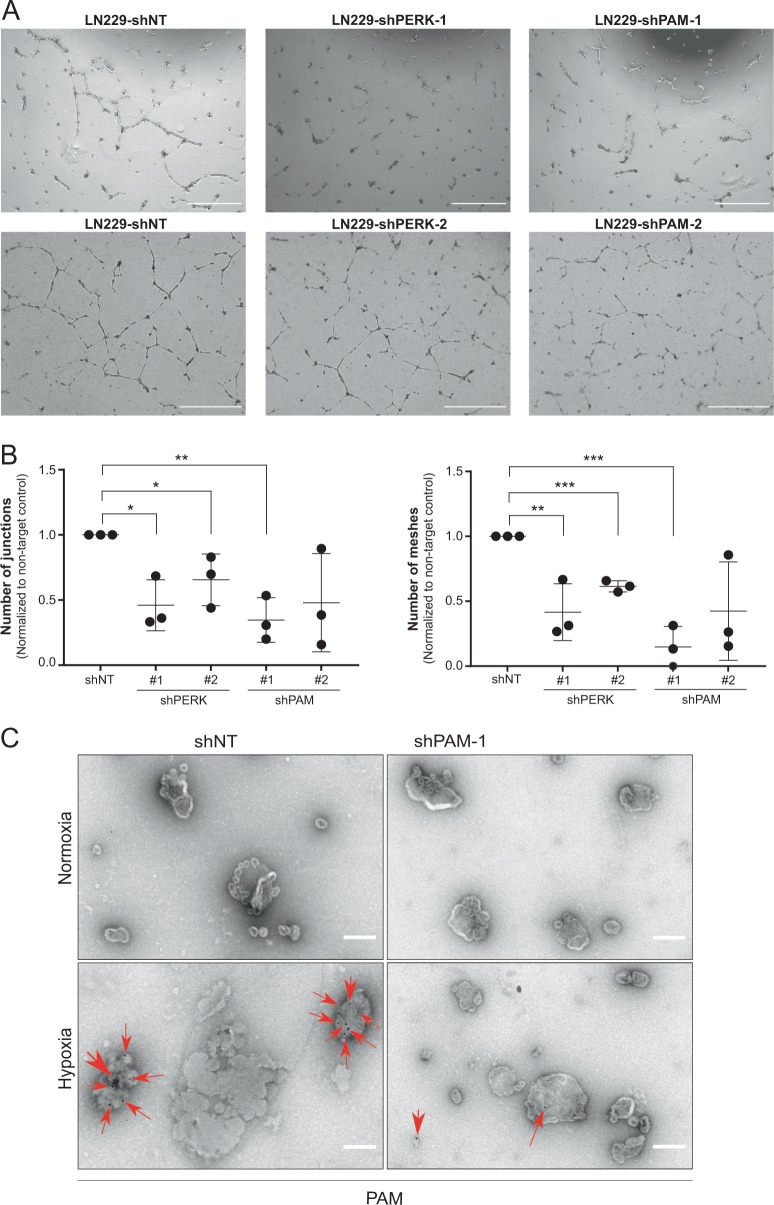
PAM expression is necessary for angiogenesis in vitro. **a** Tubes formed by HUVECs when treated with conditioned media from *PERK* and *PAM* knockdown LN229 cells using two different shRNAs along with nontarget control. Scale bars: 500 µm. **b** Plots showing number of junctions and meshes formed by HUVECs when treated with conditioned media from *PERK* and *PAM* knockdown LN229 cells. The data are normalized to the shNT control and is represented as a mean of three independent biological replicates ± SEM (*t* test with *p* value < 0.05*, <0.01** and <0.001***). **c** Vesicles isolated from the conditioned media of LN308 cells cultivated under hypoxia for 24 h. Vesicles (from cells with either shNT or shPAM-1) stained with immunolabeled gold (Au) particles for PAM detection. Red arrows indicate Au-label. Scale bars: 100 nm.

In order to determine whether PERK and PAM are essential for angiogenesis in glioblastoma, we performed the standard tube formation assay where we subjected primary human umbillical vein endothelial cells (HUVECs) with conditioned media from non-target and *PERK* or *PAM* knockdown LN229 and LN308 cells treated under hypoxia and allowed them to form tubes on growth factor-reduced matrigel within 24 h. The number of junctions and tube meshes formed by HUVECs significantly decreased when nourished with conditioned media from cells with knockdown of either *PERK* or *PAM* in comparison to non-target control (Fig. 6a, b and Supplementary Fig. S5A and B), suggesting that PAM is required for angiogenesis, even though the silencing of *PERK* might impair other secreted factors that might also be involved in this process. In line with these findings, active adrenomedullin (ADM-NH_2_) that requires PAM for its activation also increased the number of junctions and tube meshes formed by HUVECs (Supplementary Fig. S5C and D). As we have shown the secretion of PAM in conditioned media of glioblastoma cells (Fig. 2a and Supplementary Fig. S6A), the next step was to determine whether PAM is also present in extracellular vesicles from glioblastoma cells. For this, we prepared a crude extracellular fraction from the conditioned media of LN308 cells kept under hypoxia and demonstrated the expression of PAM in extracellular vesicles by immuno-electron microscopy (Fig. 6c and Supplementary Fig. S6B). We also observed reduced expression of PAM protein in extracellular vesicles isolated from PERK-silenced LN308 and LN229 cells under hypoxia in comparison to the nontarget control (Supplementary Fig. S6C).

**Fig. 7:**
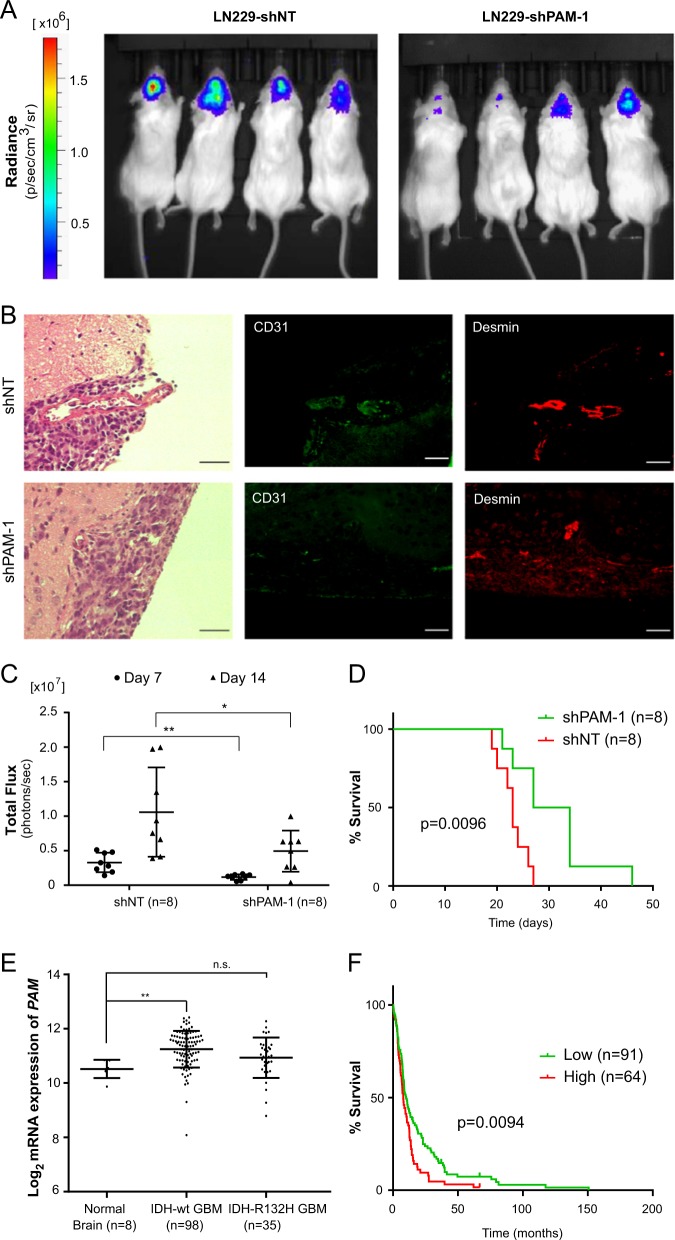
PAM expression is necessary for progression of glioblastoma in vivo. **a** Representative bioluminescence images of mice 14 days after implantation with LN229 cells expressing shNT or shPAM-1 for the indicated timepoints. **b** Representative H&E staining and immunofluorescence images for CD31 (green) and Desmin (red) of mouse tumor sections 14 days after implantation with LN229 cells expressing shNT or shPAM-1 expression. Scale bars: 50 μm. **c** Tumor growth kinetics on the basis of total flux calculated for mice implanted with LN229 cells having either shNT or shPAM-1 for the indicated timepoints. The data are normalized to respective shNT control and contains eight mice per cohort ± SD (*t* test with *p* value < 0.05*, <0.01**). **d** Overall survival of mice with shNT and shPAM-1 LN229 glioblastoma using Kaplan−Meier survival analysis. Significance was calculated using the Log-rank method. **e** Expression levels of PAM in IDH-wild-type and IDH-R132H glioblastoma patients vs. the normal brain control (data retrieved from Gravendeel et al.^44^) ± SD (*t* test with *p* value < 0.01**). **f** Kaplan−Meier survival analysis representing percentage survival of patients with low (green) and high (red) PAM expression from Gravendeel et al.^44^. Patient’s allocation to the high and low groups was such that the difference in the survival curve was as significant as possible. Significance was calculated using Log-rank method.

As PERK silencing significantly reduced cell viability of LN229 in vitro (Supplementary Fig. S7A), we determined the effect of PAM knockdown on angiogenesis, glioblastoma progression and overall survival of mice. In that respect, we implanted LN229 cells expressing luciferase with either shNT or shPAM-1 in immuno-compromised NSG mice and recorded total flux (photons/s) 7 and 14 days after implantation (Fig. 7a). CD31 and Desmin immunostaining (endothelial and pericyte marker respectively) on tissue sections from shNT and shPAM-1 mice indicates a slight effect on blood vessel formation of PAM knockdown 14 days after implantation (Fig. 7b and Supplementary Fig. S7B). Notably, reduced PAM expression significantly decreases tumor growth kinetics and increased the overall survival in the glioblastoma model (Fig. 7c, d). Interestingly, PAM expression is also increased in IDH-wild-type glioblastoma patients as compared to IDH-R132H glioblastoma patients and normal brain (Fig. 7e). It is also enhanced in the mesenchymal and classical subtype of glioblastoma (Supplementary Fig. S7C) and its high expression correlate with poor prognosis (Fig. 7f).

## Discussion

By investigating the secretome of glioblastoma, we have identified a new function of the UPR sensor PERK in the mediation of the expression and cleavage of PAM. The UPR plays a major role in cancer progression being involved in the activation or repression of oncogenes and tumor-suppressor genes such as *BRAFv600E*^11^ and *H-RAS*^2,12,13^, and the regulation of proliferation and angiogenesis^14^. It also plays a role in invasion and tumor metastasis via the regulation of transcription factors such as SNAIL1, SNAIL2, ZEB2 and TCF3^15^. Most importantly, the UPR is among the main mechanisms involved in the adaptation of glioblastoma to hypoxic stress, inducing its aggressive phenotype^3,16^. When characterizing the activation of different UPR branches in low oxygen conditions, we found a mild activation of PERK and ATF6α only, and a reduction in the activity of both domains of IRE1α. This could be a consequence of better adaptation of these cells to hypoxic stress conditions avoiding the activation of proapoptotic mechanisms downstream of UPR signaling such as CHOP production or IRE1α-mediated activation of TRAF2-JNK-ASK1 signaling^17,18^. Unlike previous studies, our data suggest a selective activation of different UPR branches under 1% O_2_ in glioblastoma, a phenomenon that could be explained by the different levels of oxygen to which the tumor cells are exposed in the different tissues^19,20^.

As PERK is known to regulate the expression of several angiogenic factors such as VEGF-A, IL6 and FGF^3,21^, we aimed to identify secreted factors that might be involved in PERK-mediated angiogenesis in glioblastoma. By proteomic screening of the secretome of the glioblastoma cell line LN308 under hypoxic stress, we identified PAM to be regulated by PERK. PAM has been shown previously to induce cytoskeleton rearrangements as well as the production and secretion of neuro-peptides^22,23^. It is composed of several luminal domains, a transmembrane domain and an unstructured cytosolic domain (PAM-CD), which facilitates its ability to interact with different proteins. The multiple phosphorylation sites among the 86 amino acid residues in this unstructured domain determine endocytic trafficking and proteolytic cleavage of PAM and influence the generation of PAM soluble fragment-cytosolic domain (sfCD)^24,25^. Like other membrane proteins, such as SREBP (Sterol-regulatory element-binding protein) or ICA512 (an autoantigen of type I diabetes), PAM sfCD is also evidenced to transfer signals to the nuclei as a feedback mechanism^26,27^. Several studies have shown the importance of PAM sfCD for tumor migration and angiogenesis. Indeed, gene expression profiling of AtT-20 cells (mouse corticotrope tumor cells) overexpressing PAM showed increased expression of Aquaporin 1 (*Aqp1*) and secretory leukocyte peptidase inhibitor (*Slpi*), affecting formation and proteome content of secretory granules^28–30^, both of which were validated to be regulated by PAM sfCD^24^. Moreover, *Aqp1* was also described to be involved in tumor migration, invasion and angiogenesis in glioblastoma^31,32^. By treating glioblastoma cells with a PERK activator, we show that its kinase activity is involved in the generation of the PAM sfCD, and the proteins directly interact. Recently published RNA-seq data showed many different pathways to be affected by PAM. Atf3, one of the major repressor transcription factors of UPR genes, was downregulated upon PAM overexpression while Fkbp2, a peptidyl prolyl isomerase (PPIase) from the FK506 binding protein (FKBP) family responsible for rate-limiting step in protein folding, was upregulated, highlighting the role of PAM in influencing ER protein folding capacity^33^. Our results, in the light of these studies, provide a strong hint towards the involvement of PAM in regulating essential pathways in glioblastoma including protein folding capacity of the ER, endocytic trafficking and secretion of factors necessary for tumor growth and angiogenesis.

Interestingly, although our data showed that PERK kinase activity has a major impact on PAM sfCD, only PERK silencing showed a very strong effect on *PAM* mRNA levels, a phenomenon that seemed to be HIF1α-dependent. Through *in silico* analysis of the PAM promoter region, we identified several putative transcription factors that might be involved in the regulation of *PAM* mRNA expression, among which was FOSL1, a component of the AP-1 complex. AP-1 consists of homo- or heterodimers of Jun and Fos family proteins and has been linked to EMT, invasion and metastasis in different cancer types including glioblastoma, colorectal and ovarian cancer^34,35^. Moreover, HIF1α is an important regulator of AP-1, driving the nuclear localization of c-jun^36^. Our data suggest an AP-1-mediated regulation of *PAM* mRNA and protein expression under hypoxic conditions, as shown by AP-1 inhibition. However, the potential involvement of other AP-1 subunits remains to be elucidated.

**Fig. 8:**
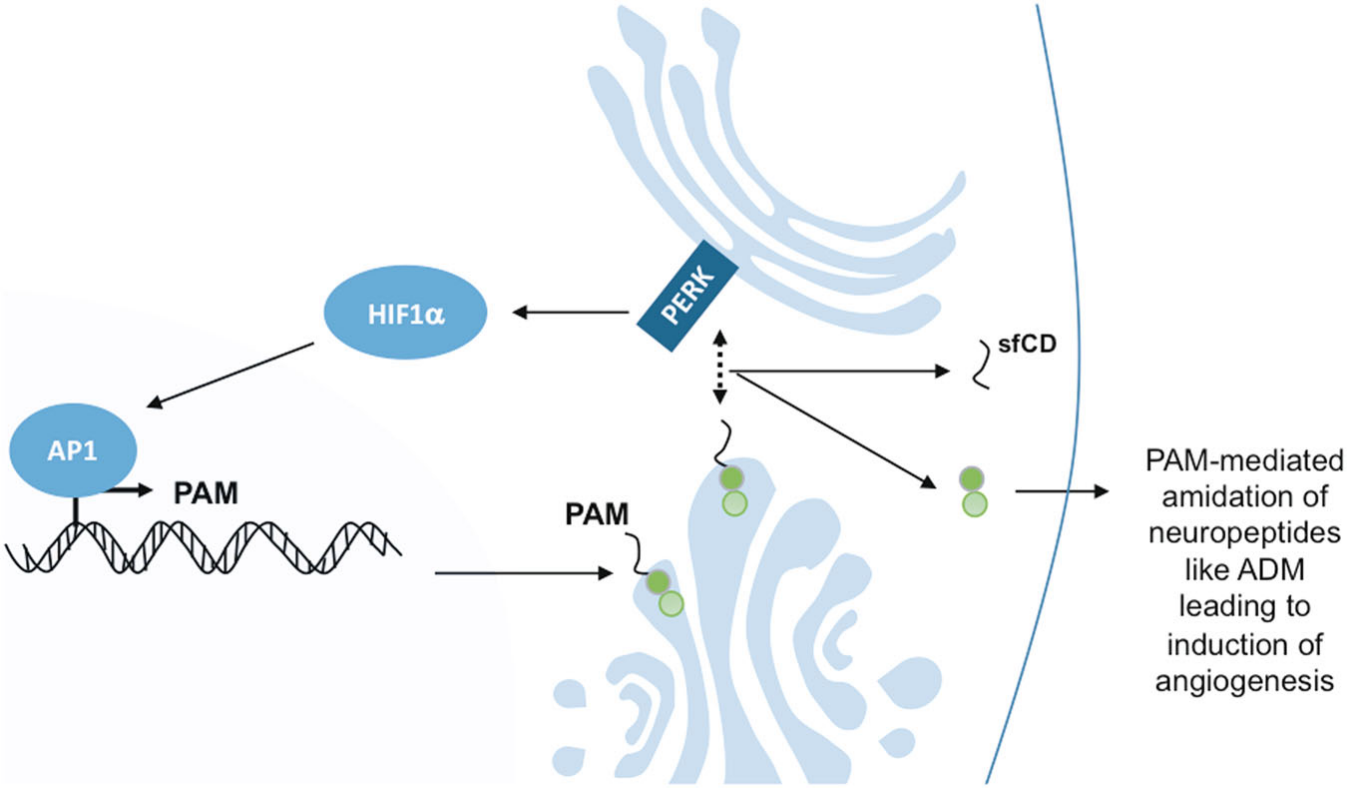
Regulation of PAM *via* PERK. Scheme showing the proposed mechanism of PERK acting on PAM mRNA expression through HIF1α and AP1 and PAM cleavage via its kinase activity.

PAM is a bi-functional type I membrane protein having monooxygenase and lyase enzymatic subunits located on its luminal domains. Several studies have shown the importance of PAM in the activation of adrenomedullin (ADM), a neuro-peptide involved in angiogenesis and invasion, as well as in reducing the proinflammatory phenotype of microglia^37–39^, and which occurred to induce tube formation in our glioblastoma cellular model. PAM homozygous knockout mice do not show any activation of ADM and die during embryogenesis due to severe edema, thinning of the aorta and carotid arteries indicating the importance of PAM in supporting ADM-mediated angiogenesis^40^. Our data highlight the role of PERK and thereby PAM in regulating angiogenesis in glioblastoma (Fig. 8).

Aberrant angiogenesis is a fundamental process in tumor development and is partly responsible for the poor prognosis of glioblastoma patients. Although they have been targeted therapeutically, inhibition of angiogenic pathways in glioblastoma has only led to marginal success in reducing tumor burden. Indeed, Bevacizumab, a monoclonal antibody targeting the proangiogenic signaling molecule VEGFA, did not show promising results in glioblastoma, improving only the progression-free survival (PFS) in patients^41^. The reasons behind the disappointing outcomes from these clinical trials may include insensitivity of anti-VEGF therapy towards tumor blood vessels^42^ and alternative angiogenic growth factor expression^43^, highlighting the importance of identifying new angiogenic factors to be targeted. Silencing PAM in glioblastoma cells reduced tumor growth kinetics in vivo and thus increased overall survival of mice, demonstrating its importance for the tumor. CD31 immunostaining of tissue section from shPAM-1 tumor in comparison to shNT tumor tissue section also hinted towards reduced blood vessel area in the tumor zone. However, a more detailed investigation is required to validate the role of PAM in glioblastoma angiogenesis in vivo. Interestingly, *PAM* mRNA expression level is significantly higher in IDH-wild-type glioblastoma patients as compared to IDH-R132H glioblastoma patients and normal brain samples and higher expression of PAM is an indicator of poor prognosis among glioblastoma patients^44^, highlighting its relevance as a therapeutic target. As PAM is significantly higher expressed in the mesenchymal and classical glioblastoma subtypes compared to the proneural subtypes, it would be important to consider patient subtype stratification in future clinical trials of agents targeting PAM.

Our study provides evidence of a new mechanism by which PERK regulates the expression and function of the proangiogenic factor PAM and highlights its potential to be considered as a therapeutic target for glioblastoma.

## Materials and methods

### Reagents

Drugs: GSK2606414 (Cay17376-1); Tunicamycin (Cay11445-10); Thapsigargin (Cay10522-1); CCT020312 (Calbiochem #324879); GSK2656157 (Cay17372-5); CoCl_2_ (C8661-25G). Primary antibodies: α-PERK (Cell Signaling, 3192S), α-eIF2α (Cell Signaling, 9722S), α-eIF2S1 (ab32157), α-ATF6α (NBP1-40256), α-IRE1α (sc-20790), α-P-IRE1α (Thermo Scientific, PA1-16927), α-EEF2 (sc-166415), α-PAM (ab-109175; recognizing the cytosolic domain), α-HIF1α (Cell Signaling, 3716S).

### Cell culture

LN308, LN229T, NCH82 glioblastoma cells were cultured in Dulbecco’s Modified Eagel Medium (DMEM) (Sigma-Aldrich, Munich, Germany) with 10% fetal calf serum (Merck-Millipore, Darmstadt, Germany) and penicillin/streptomycin (10,000 U/ml, 100 µg/ml; Thermo Fischer Scientific, Langenselbold, Germany). HUVECs were cultured in dishes precoated with 0.2% gelatin in dH_2_O. Endopan 3 medium (PAN biotech-P04-0010k) along with provided supplements was used to culture HUVECs and accutase (Sigma-Aldrich, Munich, Germany) was used to split these cells. For hypoxia treatment, cells were incubated at 1% O_2_ in a separate incubator for respective timepoints. Cells were regularly checked for mycoplasma contamination as per the recommendations of the German Collection of Microorganisms and Cells (Germany). Glioblastoma cell lines were authenticated using single nucleotide polymorphism profiling.

### Mass spectrometry experiment design

1 × 10^6^ LN308 glioma cells were seeded and were allowed to grow until 70–80% confluency (2 days). The cells were washed and incubated in serum-free DMEM. Overnight incubation of GSK2606414 (1 µM) was given as a PERK-negative control. The next day, cells were incubated under hypoxic conditions of 1% O_2._ After 72 h incubation, the conditioned media was collected in 50 ml falcon and spun down at 2000 rpm for 3 min. The conditioned media (free of debris) was collected in a new 50 ml falcon tube after filtering the content through a 0.45 µm filter. The filtered conditioned media was emptied in an Amicon Ultra-15 (PLGC Ultracel-PL Membrane, 10 kDa, UFC901008, EMD Millipore, Darmstadt, Germany). The conditioned media was concentrated by centrifuging the filter at 4000 rpm for 15 min at 4 °C. The concentrated media content was collected in a 2 ml tube and snap frozen.

### LC-MS/MS analysis

One microgram of peptide was used per injection on an Ultimate 3000 Rapid Separation Liquid Chromatography (RSLC) nano system coupled to a Q-ExactivePlus mass spectrometer (both ThermoFisher Scientific, Germany). Peptides were trapped on a precolumn (Acclaim C18 PepMap100, 100 µm 2 cm, ThermoFisher Scientific, Germany) using 0.1% trifluoroacetic acid at a flow rate of 20 µl/min and subsequently separated on a reverse phase main-column (Acclaim C18 PepMap100, 75 µm−50 cm, Thermo Scientific, Germany) using a binary gradient consisted of A: 0.1% formic acid (FA) and B: 84% acetonitrile, 0.1% FA at a flow rate of 250 nl/min. The linear gradient was run from 3 to 42% for 180 min at a flow rate of 250 nl/min. For the mass spectrometry (MS) analysis, full MS scans were acquired at a resolution of 70,000 full width at half maximum (FWHM), target value of 3e6, maximum injection time of 120 ms. Data-dependent MS scans were acquired using high-energy collisional dissociation (HCD) on 15 most abundant ions (top15) at normalized collision energy of 27%, resolution of 17,500 FWHM, isolation window of 1.2 m/z, target value of 5e4 and maximum injection time of 250 ms. Only precursor ions with charge states between 2 and 4 will be fragmented.

### Label-free data analysis

For the data analysis, Progenesis QI for Proteomics software (version 3.0 Non-Linear Dynamics) was used. The peptide identification was done using two search algorithms: X!Tandem^45^ via SearchGUI interface version 2.5.0^46^ and Mascot 2.4 (Matrix Science). The Uniprot human database (downloaded on 22nd of July 2015) was used with the following search parameters: Trypsin as protease (maximum 2 miss cleavages), fixed modification: carbamidomethylation at cysteine, variable modification: oxidation at methionine, 10 ppm as MS1 tolerance and 0.02 Da as MS2 tolerance. To combine search results from X!Tandem and Mascot, PeptideShaker version 1.9.0^47^ was used at a false discovery rate of 1%. Only proteins identified with at least two unique peptides were considered for the final analysis. The statistical data analysis was performed using R version 3.3.1. For the calculation and data formatting, packages *reshape2* were used and for the graphical illustrations *ggplot2*and*gridExtra* packages were used. The significance *p* value was calculated using function *t* test (Student’s *t* test, two-sided, true variance equality, confidence level at 0.95). Proteins were considered to be differentially regulated, if their *p* value was below 0.05 and the fold-change was greater than 2 (upregulated) or less than 0.5 (downregulated).

### Lentivirus production

Lentivirus was produced in HEK-293T cells using TransIT®-LT1 by transfecting a lentiviral construct of interest along with pMD2.G (Addgene, USA), psPAX2 (Addgene, USA). Conditioned media with the virus particles was precleared via centrifugation for 5 min at 2000 rpm and filtered through 0.45 µm cellulose acetate filters (Merck-Millipore, Germany). Lentiviral particles were harvested by centrifuging the cleared conditioned media for 90 min at 25,000 rpm at 4 °C using SW41 swing-out rotor in a L8-M ultracentrifuge. The virus particles were resuspended in PBS and stored at −80 °C. The virus titer was determined by infecting target cells with dilutions of viral particles containing the pLKO.1-puro TurboGFP plasmid (Sigma), which resulted in linear relationship between the dilution and percentage of GFP-positive cells.

### Immunoblotting

Cells were lysed in 100 µl RIPA-lysis buffer (R0278-50ML, Sigma-Aldrich, Munich, Germany) supplemented with 10 mM NaF, 10 mM Na_3_VO_4_ and complete mini protease inhibitor cocktail (Roche #11836170001, Mannheim, Germany). The lysed cells were centrifuged for 20 min at 13,000 rpm. For immunodetection of HIF1α, cells were lysed in 1% SDS/PBS and subjected to three freeze-thaw cycles in liquid nitrogen. Proteins were precipitated using 1 volume methanol and one-fourth volume of chloroform and resuspended in 5% SDS. Protein extracts were stored at −80 °C. Bicinchoninic assay was used to measure protein concentration. Protein samples were prepared using LDS sample buffer (Novex #NP0007, Life Technologies, Darmstadt, Germany) and equal amount of protein was loaded onto 4–12 % Bis-Tris Gels (40 µg from cell pellet and 3 µg from conditioned media). Electrophoresis was performed at 250 V and 170 mA for 45 min in MOPS running buffer (Life Technologies, Darmstadt, Germany). Protein was later transferred onto a polyvinylidene difluoride membrane using a protocol having subsequent increase in current every 10 min for 50 min (100, 200, 300, 400, 500 mA).The membrane was blocked, incubated with primary and subsequently with respective secondary antibody. The protein detection was done on X-ray films.

### Real-time PCR

RNA isolation was performed using Qiagen RNA isolation kit (#74106, Qiagen, Hilden, Germany) as per the product manual. One microgram of RNA was used to synthesize cDNA using ProtoScript® II Reverse Transcriptase, NEB (#M0368L, New England Biolabs (NEB), Ipswich, USA). Real-time PCR was performed by mixing 10 ng of cDNA, 0.5 µM of forward and reverse primer each (final concentration; Supplementary Table S2) and SyBr green dye (#204057, Qiagen, Hilden, Germany). A LightCycler 480 was used to perform the RT-PCR.

### PHM activity

Cells were lysed in ten volumes of buffer containing NaTES–mannitol–Triton X-100 (TMT) (pH 7.4) and protease inhibitors, as previously described^48^. Protein concentrations were determined using BCA reagent (Pierce). Samples were diluted tenfold in PHM diluent (TMT containing 1 mg/ml bovine serum albumin) and incubated at 37 °C for 1 h in the presence of 125I-Acetyl-Tyr-Val-Gly, CuSO_4_ and ascorbate (for PHM assays) or 125I-Acetyl-Tyr-Val-(OH)-Gly (for PAL assays)^48^. Uncharged amidated product was extracted with ethyl acetate and counted. Data were converted to picomoles of the product per microgram of protein per hour (pmol/μg protein/h) and analyzed with unpaired *t* tests. Each sample was assayed in triplicate.

### Immunofluorescence

The coverslips were washed with PBS twice and the cells were fixed using 4% formaldehyde overnight, washed thrice in PBS and blocked in blocking buffer (1× PBS/5% normal serum/0.3% Triton™ X-100) for 60 min. The coverslips were incubated with antibody dilution (diluted in 1× PBS/1% BSA/0.3% Triton™ X-100) for overnight at 4 °C (PERK ab (AF3999-SP, Novus Biologicals, Littleton, USA)), GM130 ab (#610822, BD Biosciences, US), Calnexin ab (#AD1-SPA-860-D, EnzoLifeSciences GmbH, Germany)), rinsed thrice with PBS and incubated with Fluorochrome-conjugated secondary antibody for 1–2 h at room temperature in the dark and humid environment. Anti-goat (ab150089, Abcam, Cambridge, UK), anti-rabbit (a10042, ThermoFisher Scientific, Massachusetts, US) and anti-mouse (ab150113, Abcam, Cambridge, UK) secondary antibodies were used having Alexa488, Alexa568 and Alexa488 labels respectively. After secondary antibody incubation the coverslips were mounted with Vectorsheild DAPI-mount (#H-1200) onto a glass slide. The images were taken using a Leica SP8 confocal microscope.

### Immunoprecipitation

Cells were lysed using Pierce IP-lysis buffer (#87787, Thermo Fischer Scientific, Langenselbold, Germany). The supernatant was collected in new tubes and precleared using 25 µl of Dynabeads Protein G (Invitrogen #10003D) for an hour at 4 °C. In parallel, 25 µl of Dynabeads was washed with PBS-Tween20 (0.1%) and incubated with 1 µg of antibody for 20 min at room temperature on a rocker in 200 µl PBS-Tween20 (0.1%). Later, 500 µg of protein from the precleared lysate was incubated with the Dynabeads-Antibody complex making a final volume of 500 µl in IP-lysis buffer for overnight incubation at 4 °C. The Dynabeads were washed with 500 µl PBS-Tween20 (0.1%) thrice and boiled at 95 °C for 4 min after resuspending in 5× Laemmli buffer and loaded onto an SDS-PAGE gel.

### Tube formation assay

As described previously, the tube formation assay was performed by coating wells of a 48-well plate with 150 µl of growth factor-reduced matrigel (Product #356230; Corning Inc., Corning, USA) and allowed to polymerize for 10 min at 37 °C ^49^. HUVECs were detached using accutase and resuspended at a concentration of 80,000 cells per 800 µl. 250 µl of cell suspension was added to each well coated with matrigel and the plate was incubated at 37 °C and 5% CO_2._ The tubes formed by HUVECs were imaged using an Axio Vert.A1microscope. The quantification was done manually. A junction is identified as a point where more than two segments meet whereas a mesh is identified as a closed network formed by the segments. A segment is a tube formed between two different junctions.

### Animal experiments

Anesthetized male NSG mice (NOD SCIDγ Jackson, USA), 10–12 weeks of age, were given an analgesic and xenografted with 8 × 10^4^ LN229 glioma cells expressing GFP and luciferase with either shNT (*n* = 8) or shPAM-1 (*n* = 8) stably integrated in the cells using a pLKO lentiviral backbone, at a position 1 mm lateral and 2 mm posterior from the *bregma*. Mice with neurological symptoms or a weight loss of >20% were euthanized. All animal procedures were conducted in accordance with the institutional animal research guidelines after obtaining approval from the regional commission of Karlsruhe, Baden-Wuerttemberg, Germany (file number G127-16). Survival was compared using the log-rank test. As it was not required for our kind of experiment, no randomization of the groups was involved. The investigator determining the endpoint was blinded.

### Bioluminescence imaging

All mice were imaged at days 7 and 14 post tumor cell injection. d-luciferin was injected at a concentration of 150 mg/kg body weight (Biomol, Germany). Mice were anaesthetized using 1.5% of isoflurane (Abbott, Germany). After 10 min incubation, images were acquired using an IVIS Lumina II system (Caliper Life Science, USA) with exposure times of 1, 3 and 5 min. Total bioluminescence flux signals (photons/s) were quantified with LivingImage 4.4 (PerkinElmer, USA) as a measure of tumor burden.

### Statistics

Excel 2010 and GraphPad Prism 7 were used to carry out statistical testing. Experiments were performed in biological triplicate (unless otherwise mentioned) and data provided in the manuscript represent the mean ± standard error or standard deviation. Unpaired two-sided Student’s *t* test was used to determine the level of significance. Log-rank (Cox−Mantel) test was used to calculate the difference in the distribution of survival data in Kaplan−Meier analysis.

## Supplementary information

Supplementary Informations

Supplementary Figure S1

Supplementary Figure S2

Supplementary Figure S3

Supplementary Figure S4

Supplementary Figure S5

Supplementary Figure S6

Supplementary Figure S7

## Data Availability

Mass spectrometry dataset generated and analyzed in this study is available on ProteomeXchange:PRIDE PXD012523.
