# Supplementary material for: PERK-mediated expression of peptidylglycine α-amidating monooxygenase supports angiogenesis in glioblastoma
Source: Oncogenesis. 2020 Feb 13;9(2):18. doi: 10.1038/s41389-020-0201-8 (PMC7018722; doi:10.1038/s41389-020-0201-8)
Supplement: Supplementary file 1 — Supplementary Informations [file 41389_2020_201_MOESM1_ESM.pdf]

# PERK-mediated expression of peptidylglycine α-amidating monooxygenase supports angiogenesis in glioblastoma

Himanshu Soni[1,2], Julia Bode[2], Chi D L Nguyen[3], Laura Puccio[1], Michelle Neßling[4], Rosario M. Piro[5,6,7], Jonas Bub[1], Emma Phillips[1], Robert Ahrends[3,8], Betty A. Eipper[9], Björn Tews[2] & Violaine Goidts[1*]

*Corresponding Author

## Supplementary Data Summary

## Table of Contents:

I. **Supplementary Tables**
II. **Supplementary Material and Methods**

**Supplementary Table S1:** Log2- and z-transformed TBAs, for 579 TF binding motifs from JASPAR, for the promoter of PAM transcripts (NM_138821). Only motifs with scores >= 1.5 are reported.

| ID | Name | z-Score |
|---|---|---|
| **MA0665.1** | MSC | 2.68 |
| **MA0784.1** | POU1F1 | 2.42 |
| **MA0787.1** | POU3F2 | 2.36 |
| **MA0786.1** | POU3F1 | 2.27 |
| **MA0866.1** | SOX21 | 2.21 |
| **MA0735.1** | GLIS1 | 2.10 |
| **MA0737.1** | GLIS3 | 2.03 |
| **MA0795.1** | SMAD3 | 2.01 |
| **MA0667.1** | MYF6 | 2.01 |
| **MA0031.1** | FOXD1 | 1.82 |
| **MA0157.2** | FOXO3 | 1.81 |
| **MA0080.4** | SPI1 | 1.80 |
| **MA0845.1** | FOXB1 | 1.77 |
| **MA0785.1** | POU2F1 | 1.73 |
| **MA0842.1** | NRL | 1.72 |
| **MA1103.1** | FOXK2 | 1.70 |
| **MA1152.1** | SOX15 | 1.67 |
| **MA0032.2** | FOXC1 | 1.63 |
| **MA0788.1** | POU3F3 | 1.61 |
| **MA0852.2** | FOXK1 | 1.61 |
| **MA0089.1** | MAFG::NFE2L1 | 1.60 |
| **MA0751.1** | ZIC4 | 1.57 |
| **MA1137.1** | FOSL1::JUNB | 1.57 |
| **MA0789.1** | POU3F4 | 1.55 |
| **MA1128.1** | FOSL1::JUN | 1.55 |
| **MA1141.1** | FOS::JUND | 1.54 |
| **MA0792.1** | POU5F1B | 1.52 |
| **MA0613.1** | FOXG1 | 1.51 |

*ID = identifier of the TF matrix.

**Supplementary Table S2:** List of primers used for quantitative PCR

| Name | Forward Primer | Reverse Primer |
|---|---|---|
| **XBP1s** | TGAGTCCGCAGCAGGTGCA | CTGGGTCCTTCTGGGTAGACCTC |
| **NDRG1** | GTTTCCTGGCGTCGTCTC | ATGTCCCTGCTGTCACCTG |
| **XBP1-EP** | CCTGGTTGCTGAAGAGGAGG | CCATGGGGAGATGTTCTGGAG |
| **PAM** | TTACACCTCACACGTCTGCC | ATCAACTGGATGCCCCACAG |
| **VEGF-A** | GGCCTCCGAAACCATGAACT | TGGGACTTCTGCTCTCCTTCT |
| **HIF1α** | TCCATGTGACCATGAGGAAA | CCAAGCAGGTCATAGGTGGT |
| **PERK** | GCCAATGACAGTAGCTGGAATG | GTGTTCAAGCTTGGCTAAGGCTT |
| **EEF2** | CTGGAGATCTGCCTGAAGGA | GAGACGACCGGGTCAGATT |
| **RPS13** | CCCAGTCGGCTTTACCCTAT | GCCCTTCTTGGCCAGTTTGT |

**Supplementary Table S3:** List of shRNA target sequences

| Target Gene | Target sequence |
|---|---|
| **PERK-1** | GGCAACCATTGTGCTAATAAA |
| **PERK-2** | GCCACTTTGAACTTCGGTATA |
| **HIF1α** | CCGCTGGAGACACAATCATAT |
| **PAM** | GCCTTTAATTGCTGGCATGTA |
| **Non-Target (NT)** | CAACAAGATGAAGAGCACCAA |

## Supplementary Material and Methods

### pLKO.1 shRNA Cloning

shRNA oligos (Supplementary Table S3) were primed at 95°C for 4 min using hybridization buffer (TrisHCl (pH 7.8), 100 mM, NaCl 1M, and EDTA 10 mM). The primed oligos were then ligated into pLKO.1 vector digested with EcoRI and AgeI restriction enzymes using T4 DNA ligase (EL0011) overnight. The ligation mix was transformed in *E. Coli* DH5α competent cells and colonies were confirmed for the presence of shRNA oligos first using colony screening PCR and later sequencing.

### Proteomics sample preparation

Each sample type was prepared in three biological replicates. For the protein precipitation, 2.25 ml of ice cold ethanol was added to 250 µl of cell secretome samples. After ~16 hour of precipitation at -80°C, the protein pellets were collected by centrifugation at 18.000 g, 4 °C for 30 min. 20 µl of 6M Guanidine-Hydrochloride (Gu-HCl, Sigma-Aldrich, USA) was used to dissolve the protein pellets. 50 mM of $NH_4HCO_3$ (pH 7.8, Sigma-Aldrich, USA) was added to dilute the concentration of Gu-HCl to 0.6 M for the protein amount determination using bicinchoninic acid assay (Pierce™ BCA Protein Assay Kit, ThermoFisher Scientific™ Hamburg Germany). The samples were then reduced with 10 mM final concentration of Tris (2-carboxyethyl) phosphine hydrochloride (TCEP, Sigma-Aldrich, USA) at 56 °C for 30 min, alkylated with 30 mMIodoacetamide (IAA, Sigma-Aldrich, USA) at 25 °C in dark chamber for 30 min and diluted to 0.2 M Gu-HCl with 50 mM of $NH_4HCO_3$ (pH 7.8) for Trypsin digestion. Trypsin (Trypsin Gold, Promega, USA) was added at a ratio of 1:50 (w/w, protease to substrate) for the enzymatic digestion. The digestion was performed for maximum 15 hours at 37 °C. The digestion was stopped by acidifying with 10% trifluoroacetic acid (TFA) until pH <2. The peptide samples were desalted using solid phase extraction method utilizing SepPak C18 cartridges (100 mg sorbent per cartridge, Waters, USA). The cartridges were preconditioned twice with 1 ml methanol each time and then equilibrated three times with 0.1% TFA. The peptide samples were loaded onto the cartridges. After washing three times

with 1 ml 0.1% TFA, they were eluted with 80% acetonitrile (ACN), 0.1% formic acid (FA) and dried under vacuum. The dried peptides were dissolved in 0.1% TFA and stored at -80°C for further analysis. The digestion quality was checked by monolithic reverse phase separation as described in Burkhart et al. 2012.

**Extracellular vesicle isolation**

The conditioned media was collected and taken through sequential centrifugation steps in order to obtain clean extracellular vesicle fraction. The conditioned media was first centrifuged at 1500 rpm for 10 min to remove cell debris. Supernatant was collected and centrifuged at 2500 rpm for 10 min and 4000 rpm for 30 min. The supernatant was used in each step for further processing. The final step involved the centrifugation of collected supernatant at 25000 rpm for 3 hours to obtain extracellular vesicles. The supernatant was discarded and pellet obtained was either resuspended in RIPA for western blotting or reconstituted in PBS overnight at 4°C for electron microscopy imaging.

**Electron Microscopy**

For electron microscopy (EM) extracellular vesicles from purified preparations were adsorbed onto glow discharged carbon coated formvar grids, washed in buffer and negatively stained with 2% aqueous uranyl acetate. For immuno-EM, the immuno reaction was performed after buffer wash including incubation with blocking agent (Aurion, Wageningen, The Netherlands), dilution series of primary antibody and Protein A-Au reporter (CMC, UMC Utrecht, The Netherlands). Micrographs were taken with a Zeiss EM 910 at 100kV (Carl Zeiss, Oberkochen, Germany) using a slow scan CCD camera (TRS, Moorenweis, Germany).

**TCA protein precipitation**

Conditioned media was collected and spun down to remove cell debris. The supernatant was then mixed with trichloroacetic acid (TCA) making a final concentration of 10%, and kept at

4°C overnight on ice. The tubes were centrifuged at 13000 rpm for 30 min to pellet down the precipitated protein. Supernatant was discarded and the pellets were washed twice with acetone (kept at -20°C) by centrifuging at 13000 rpm for 30 min. Later, the pellet was dried for two minutes to remove excess of acetone and re-suspended in RIPA buffer. Equal amount of protein was loaded for SDS-PAGE.

**Nuclear-Cytoplasmic Fractionation**

Cells were lysed in PBS + 0.1% NP40 containing protease & phosphatase inhibitors for 2 min on ice and centrifuged at 6500 rpm for 1 min at 4°C. The supernatant was collected as cytoplasmic fraction while the pellet is washed twice in PBS + 0.1% NP40. Later, the nuclear pellet is lysed in RIPA buffer (R0278-50ML, Sigma-Aldrich, Munich, Germany) supplemented with 10 mMNaF, 10 mM $Na_3VO_4$ and complete mini protease inhibitor cocktail (Roche #11836170001, Mannheim, Germany).

**Immunohistochemistry**

Mice were sacrificed by CO2 asphyxiation. Brains were extracted and perfused with 10 % (w/v) formaldehyde. They were then sectioned coronally, dehydrated in a STP 120 spin tissue processor and embedded in paraffin. Sections of 4 µm thickness were cut and mounted onto glass slides, deparaffinised in xylene and rehydrated in a descending series of alcohols. Slides were treated with primary antibodies (CD31 #AF3628; Desmin #ab15200-1) overnight at 4°C and incubated with a HRP conjugated secondary antibody (#a21206 and #a21085) for 1 h at room temperature. Staining was visualised using the Dako REAL Detection System and freshly prepared diaminobenzidine as a chromogen. Slides were counterstained with haematoxylin, dehydrated and mounted. Images were made using a Zeiss Axioplan 2 microscope. Pictures were taken with a 40x objective, fixed intensity and gain settings and images were analyzed using Fiji. Different tumor zones were imaged from

1 mouse per cohort sacrificed after 14 days of glioblastoma cells implantation. CD31 and Desmin-positive area were correlated to the total tumor area per image.

**Total binding affinity analysis for PAM promoters**

Genome-wide total binding affinities (TBA) for transcription factors of all promoters of protein-coding genes was performed as follows: Curated gene transcript annotation was downloaded (December 20, 2018) from the RefSeq track of the UCSC Genome Browser (human reference genome GRCh38/hg38)[2] and filtered for transcripts which contain coding sequences. A promoter was defined as the region spanning from 1500 bases upstream to 500 bases downstream (with respect to the transcription strand) of the transcription start site (TSS). For genes with multiple TSS, all promoters were considered individually, resulting in a total of 25,387 promoters of 19,301 protein-coding genes. Position frequency matrices (PFM) were obtained from JASPAR (release 7, 2018)[3] for core matrices of a total of 579 vertebrate TF, using the R package JASPAR2018 (version 1.1.1) (https://bioconductor.org/packages/release/data/annotation/html/JASPAR20). For each promoter and each TF, we computed the binding affinity using the R package MatrixRider (version 1.12.0) (https://bioconductor.org/packages/release/bioc/html/MatrixRider.html) with a cutoff of zero for *getSeqOccupancy()*, corresponding to the TBA, i.e., the binding affinity summed over the entire promoter rather than over individual TF binding sites[4]. The resulting set of 25,387 TBA obtained for each TF was transformed first by taking the logarithm (base 2) and then by computing the corresponding z-score (i.e., the number of standard deviations it is above or below the mean log2-transformed TBA for that TF). This transformation allows for better comparability between TBA of different TF because the raw TBA scores highly depend on both the length and the information content of the different binding motifs.

## References

1 Burkhart JM, Schumbrutzki C, Wortelkamp S, Sickmann A, Zahedi RP. Systematic and quantitative comparison of digest efficiency and specificity reveals the impact of trypsin quality on MS-based proteomics. *J Proteomics* 2012; **75**: 1454–1462.

2 Kent WJ *et al.* The human genome browser at UCSC. *Genome Res* 2002; **12**: 996–1006.

3 Khan A *et al.* JASPAR 2018: update of the open-access database of transcription factor binding profiles and its web framework. *Nucleic Acids Res* 2018; **46**: D260–D266.

4 Grassi E, Zapparoli E, Molineris I, Provero P. Total Binding Affinity Profiles of Regulatory Regions Predict Transcription Factor Binding and Gene Expression in Human Cells. *PLoS One* 2015; **10**: e0143627.
