# Supplementary material for: PERK-mediated expression of peptidylglycine α-amidating monooxygenase supports angiogenesis in glioblastoma
Source: Oncogenesis. 2020 Feb 13;9(2):18. doi: 10.1038/s41389-020-0201-8 (PMC7018722; doi:10.1038/s41389-020-0201-8)
Supplement: Supplementary file 2 — Supplementary Figure S1 [file 41389_2020_201_MOESM2_ESM.pdf]

Figure S1

A

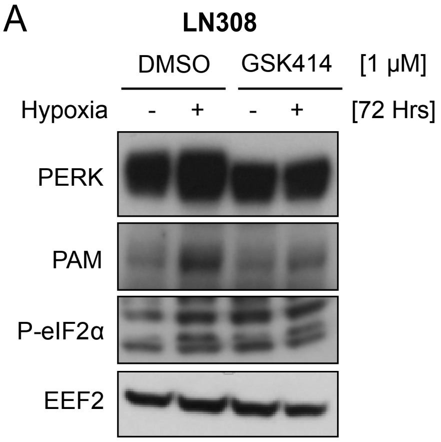

Supplementary Figure S1. GSK2606414 reduces PERK activation in LN308 cells. **A)** Levels of PERK, PAM, and P-eIF2α protein in LN308 glioblastoma cells treated with PERK-specific inhibitor GSK2606414 under hypoxia for 72 hours. EEF2 was used as a loading control.
