# Supplementary material for: PERK-mediated expression of peptidylglycine α-amidating monooxygenase supports angiogenesis in glioblastoma
Source: Oncogenesis. 2020 Feb 13;9(2):18. doi: 10.1038/s41389-020-0201-8 (PMC7018722; doi:10.1038/s41389-020-0201-8)
Supplement: Supplementary file 3 — Supplementary Figure S2 [file 41389_2020_201_MOESM3_ESM.pdf]

Figure S2

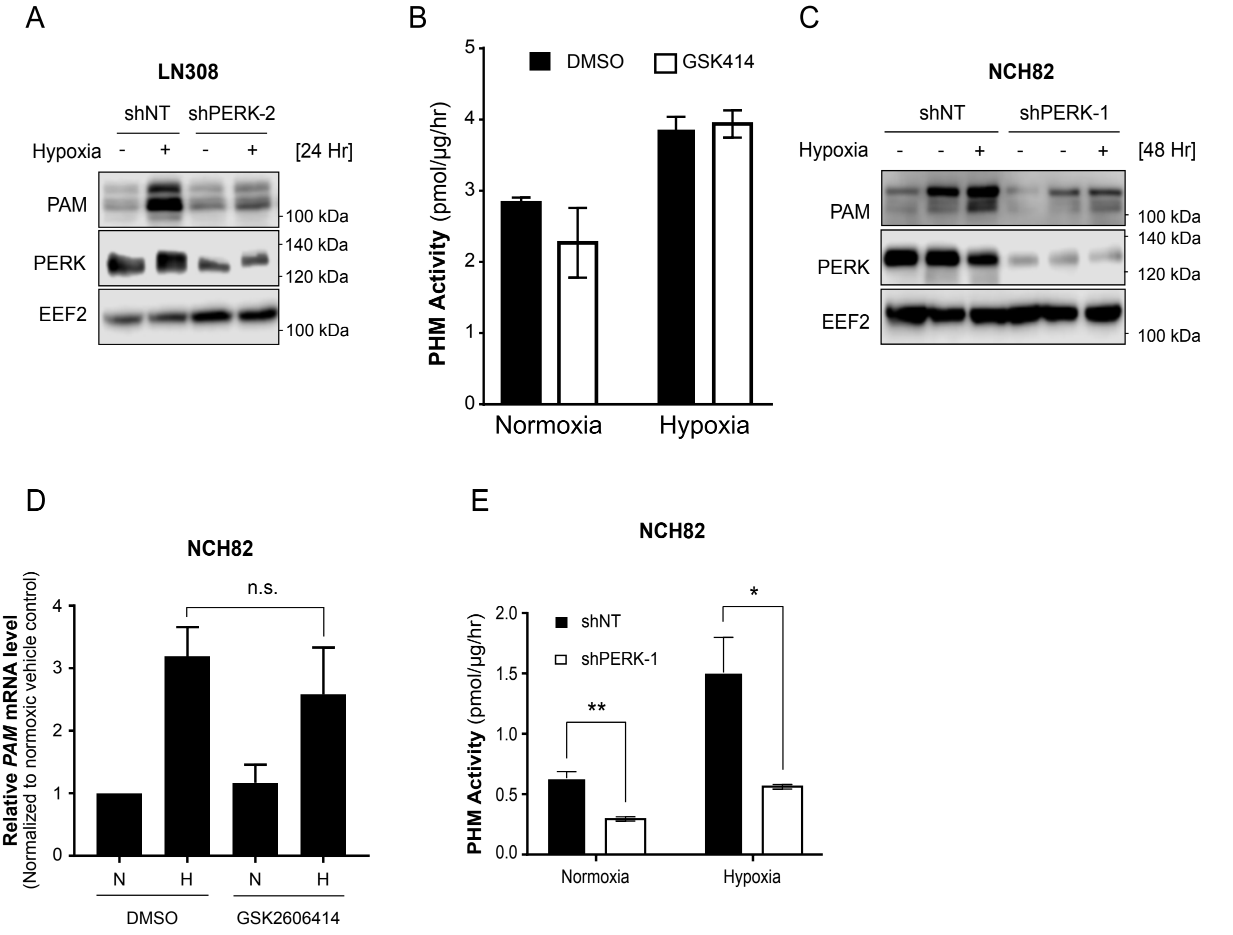

Supplementary Figure S2. PERK regulates PAM on the mRNA level independent of the PERK kinase. A) PAM protein expression in LN308 cells upon PERK silencing using shPERK-2 under hypoxia for 24 hours. **B)** PHM activity was quantified from LN229 cells with PERK kinase inhibition under hypoxia for 24 hours. The data are represented as the mean of three independent experiments ± SEM. GSK414 represents GSK2606414. **C)** Expression of PAM under *PERK* knockdown in NCH82 low passage patient derived glioblastoma cells under hypoxia for 48hours. **D)** Relative *PAM* mRNA levels in NCH82 cells under PERK kinase inhibition using GSK2606414 (500 nM) when treated with hypoxia for 24 hours (mean of three independent replicates ± SEM; n.s.: not significant). N-Normoxia, H-Hypoxia. **D)** PHM activity in NCH82 cells under hypoxia for 24 hours upon *PERK* knockdown. The data are represented as the mean of three independent biological replicates ± SEM (t-test with p-value < 0.05* and < 0.01**).
