# Supplementary material for: PERK-mediated expression of peptidylglycine α-amidating monooxygenase supports angiogenesis in glioblastoma
Source: Oncogenesis. 2020 Feb 13;9(2):18. doi: 10.1038/s41389-020-0201-8 (PMC7018722; doi:10.1038/s41389-020-0201-8)
Supplement: Supplementary file 4 — Supplementary Figure S3 [file 41389_2020_201_MOESM4_ESM.pdf]

Figure S3

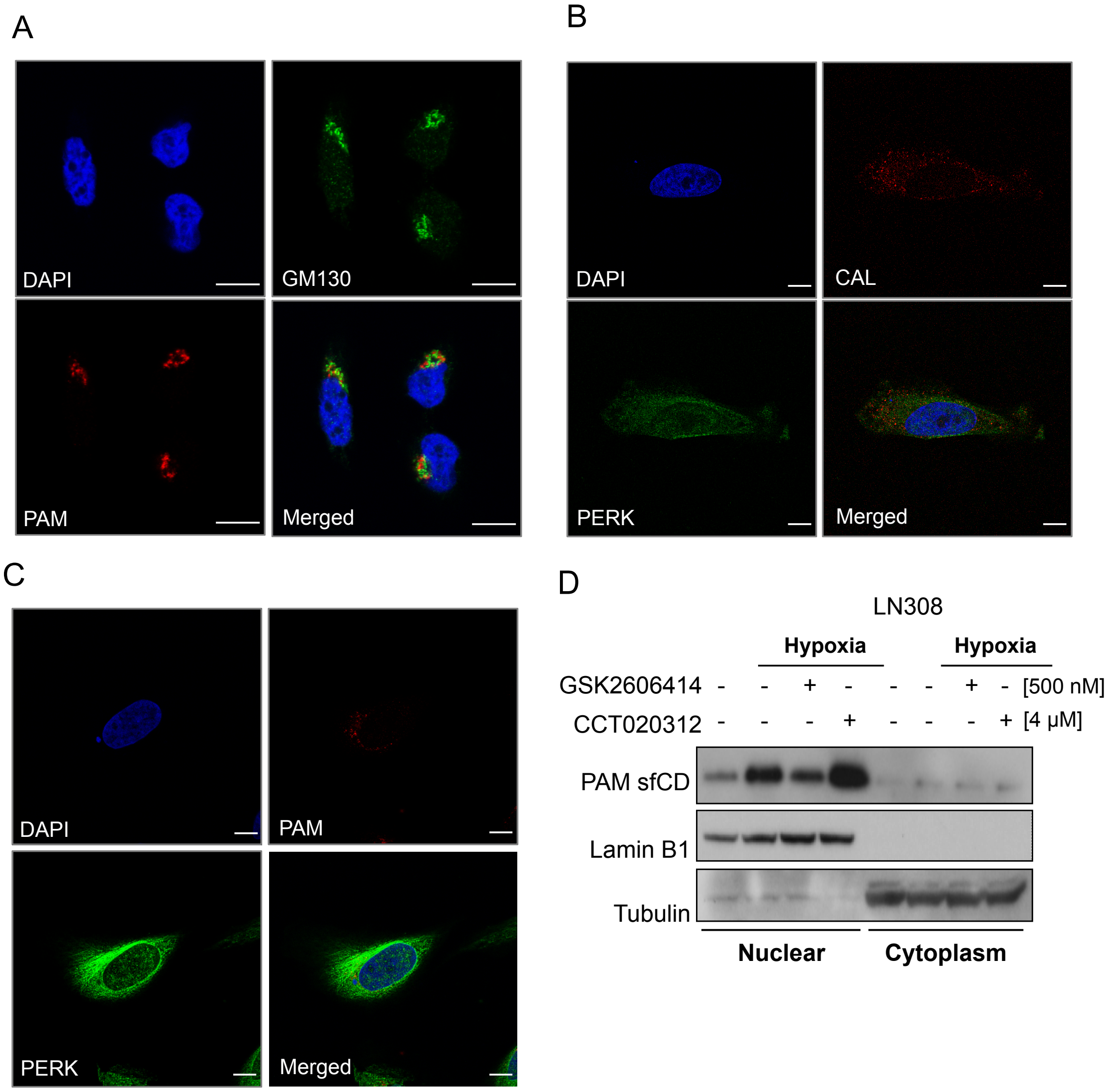

Supplementary Figure S3. Localization and regulation of PAM in glioblastoma. Immunofluorescence images of **A)** PAM and GM130 (Golgi marker), and **B)** PERK and Calnexin (ER marker) from LN229 cells treated with hypoxia for 24 hours. DAPI was used as a nuclear stain. Scale bars: 10 μm. **C)** PAM and PERK immunofluorescence images from LN229 cells under normoxia. DAPI was used as nuclear stain. Scale bars: 10 μm. **D)** Localization of PAM sfCD under the influence of CCT020312-mediated PERK activation and GSK2606414-mediated inactivation of PERK in LN308 cells cultivated under hypoxia for 24 hours. LaminB1 was used as a loading control for the nuclear fraction and α-tubulin was used as a loading control for the cytoplasmic fraction.
