# Supplementary material for: PERK-mediated expression of peptidylglycine α-amidating monooxygenase supports angiogenesis in glioblastoma
Source: Oncogenesis. 2020 Feb 13;9(2):18. doi: 10.1038/s41389-020-0201-8 (PMC7018722; doi:10.1038/s41389-020-0201-8)
Supplement: Supplementary file 5 — Supplementary Figure S4 [file 41389_2020_201_MOESM5_ESM.pdf]

Figure S4

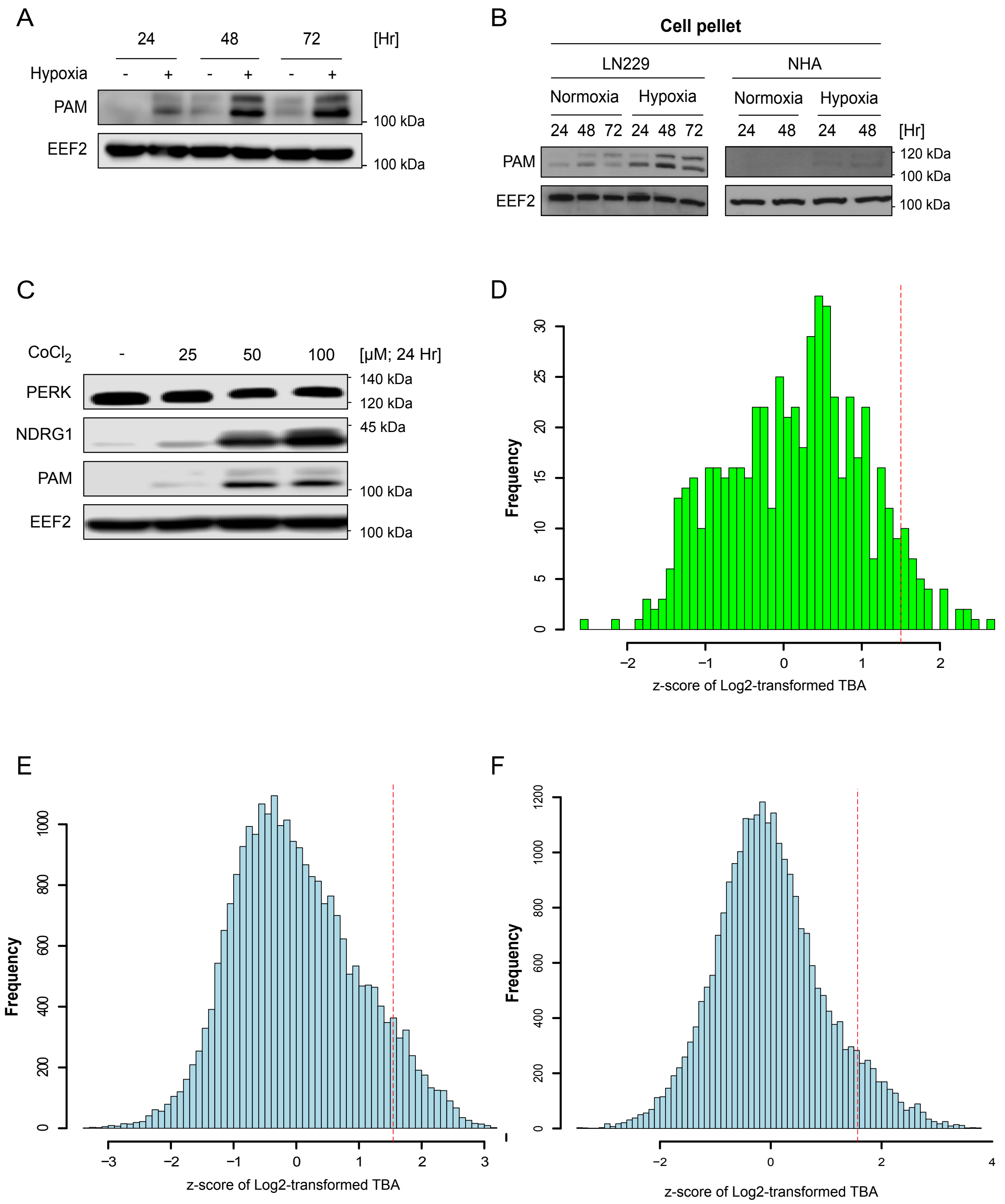

Supplementary Figure S4. Total binding affinities of the PAM promoter. A) Expression of PAM in LN308 glioblastoma cell line over time in normoxia and hypoxia. EEF2 was used as a loading control. **B)** Expression of PAM under hypoxia in LN229 and human astrocytes (NHA). EEF2 was used as a loading control. **C)** PAM protein levels under $CoCl_2$ treatment in LN308 cells. NDRG1 was used as a marker of HIF1α and EEF2 was used as a loading control. **D)** Distribution of log2- and z transformed TBAs of all 579 TF binding motifs on the promoter of PAM transcripts NM_138821.The red line indicates a z-score of 1.5 indicating a log2-transformed TBA of half a standard deviation above the average. Distribution of log2- and z-transformed TBAs of FOSL1::JUN **(E)** and FOSL1::JUNB **(F)** for the 25,387 promoters. The red vertical line indicates the z-score of both the transcription complex pair obtained for the PAM promoter.
