# Supplementary material for: PERK-mediated expression of peptidylglycine α-amidating monooxygenase supports angiogenesis in glioblastoma
Source: Oncogenesis. 2020 Feb 13;9(2):18. doi: 10.1038/s41389-020-0201-8 (PMC7018722; doi:10.1038/s41389-020-0201-8)
Supplement: Supplementary file 6 — Supplementary Figure S5 [file 41389_2020_201_MOESM6_ESM.pdf]

Figure S5

A

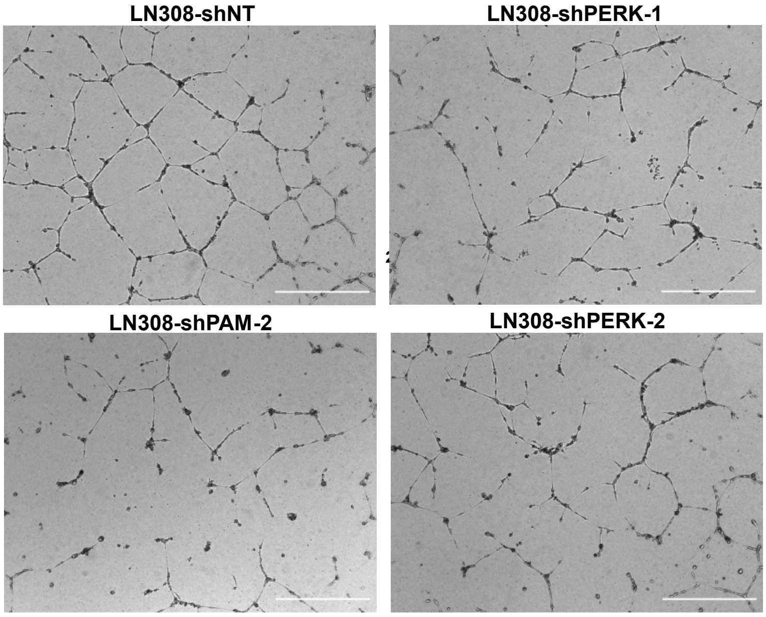

B

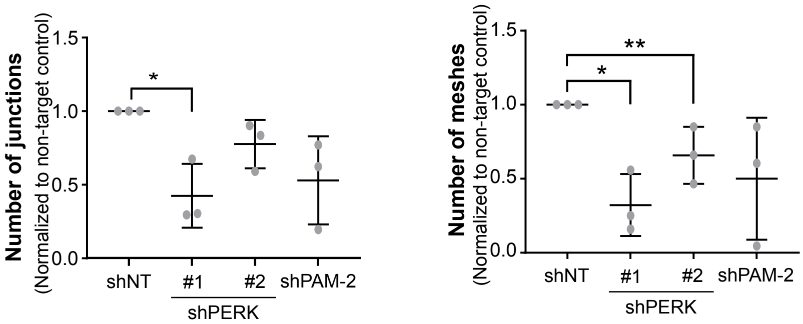

C

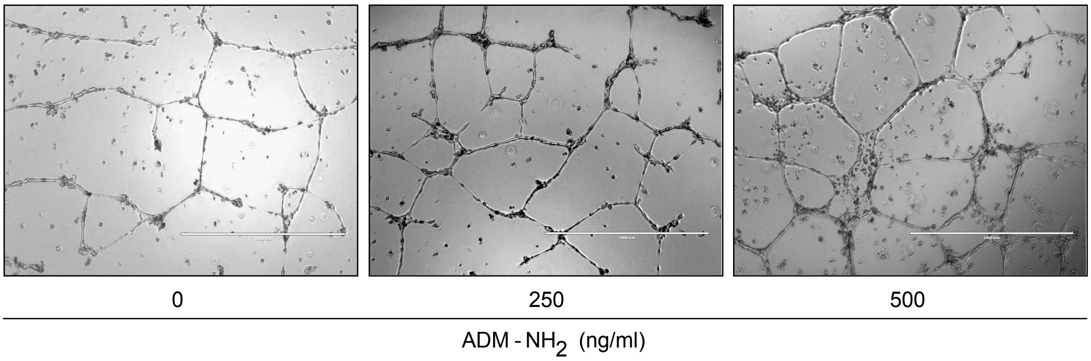

D

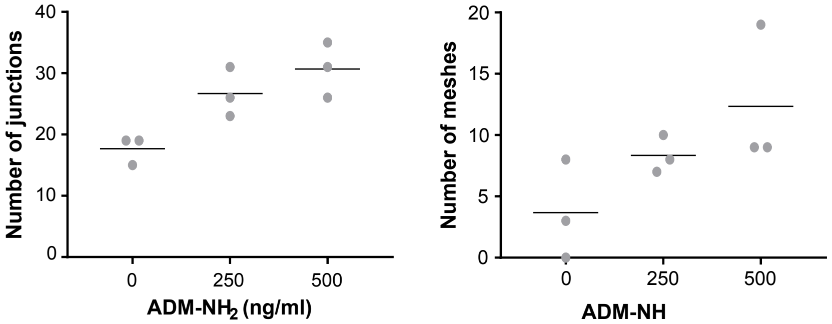

Supplementary Figure S5. PAM expression is necessary to induce tube formation by HUVECs *in vitro*. A) Tubes formed by HUVECs when treated with conditioned media from *PERK* and *PAM* knockdown LN308 cells. Scale bars: 500 µm. **B)** Plots showing number of junctions and meshes formed by HUVECs when treated with conditioned media from *PERK* and *PAM* knockdown LN308 cells. The data is normalized to the shNT control and is represented as the mean of three independent biological replicates ± SEM. **C)** Images showing tubes formed by HUVECs over reduced-matrigel for 18 hours in the presence of different concentrations of active adrenomedullin. The data are represented as the mean of three technical replicates. Scale bars: 1000 µm. **D)** Corresponding numbers of junctions and meshes formed by HUVECs under adrenomedullin treatment.
