# Supplementary material for: PERK-mediated expression of peptidylglycine α-amidating monooxygenase supports angiogenesis in glioblastoma
Source: Oncogenesis. 2020 Feb 13;9(2):18. doi: 10.1038/s41389-020-0201-8 (PMC7018722; doi:10.1038/s41389-020-0201-8)
Supplement: Supplementary file 7 — Supplementary Figure S6 [file 41389_2020_201_MOESM7_ESM.pdf]

Figure S6

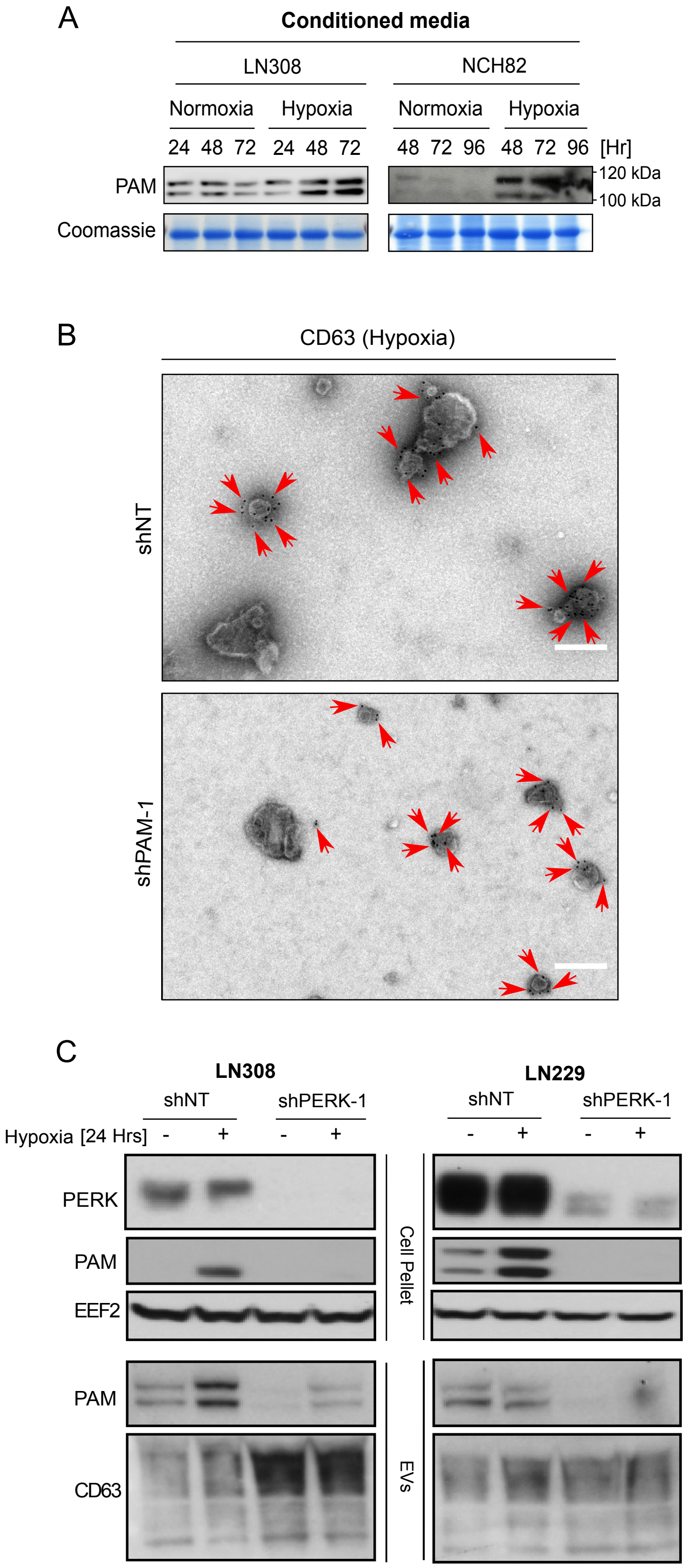


**Supplementary Figure S6. PAM is also secreted in extracellular vesicles from LN308 glioblastoma cells under hypoxia. A)** Secretion of PAM protein in conditioned media from LN308 and NCH82 cells. **B)** Vesicles isolated from the conditioned media

of LN308 cells cultivated under hypoxia for 24 hours. Vesicles stained with immuno-labeled gold (Au) particle to detect CD63(an exosomal marker) from cells with either shNT or shPAM-1. Red arrows indicate Au-label. Scale bars: 100 nm. **C)** PAM expression in extra-cellular vesicles isolated from the conditioned media of LN308 and LN229 glioblastoma cells expressing shNT or shPERK-1 when treated with hypoxia for 24 hours. EEF2 and CD63 were used as loading control for cells and extra-cellular vesicles, respectively.
