# Supplementary material for: PERK-mediated expression of peptidylglycine α-amidating monooxygenase supports angiogenesis in glioblastoma
Source: Oncogenesis. 2020 Feb 13;9(2):18. doi: 10.1038/s41389-020-0201-8 (PMC7018722; doi:10.1038/s41389-020-0201-8)
Supplement: Supplementary file 8 — Supplementary Figure S7 [file 41389_2020_201_MOESM8_ESM.pdf]

Figure S7

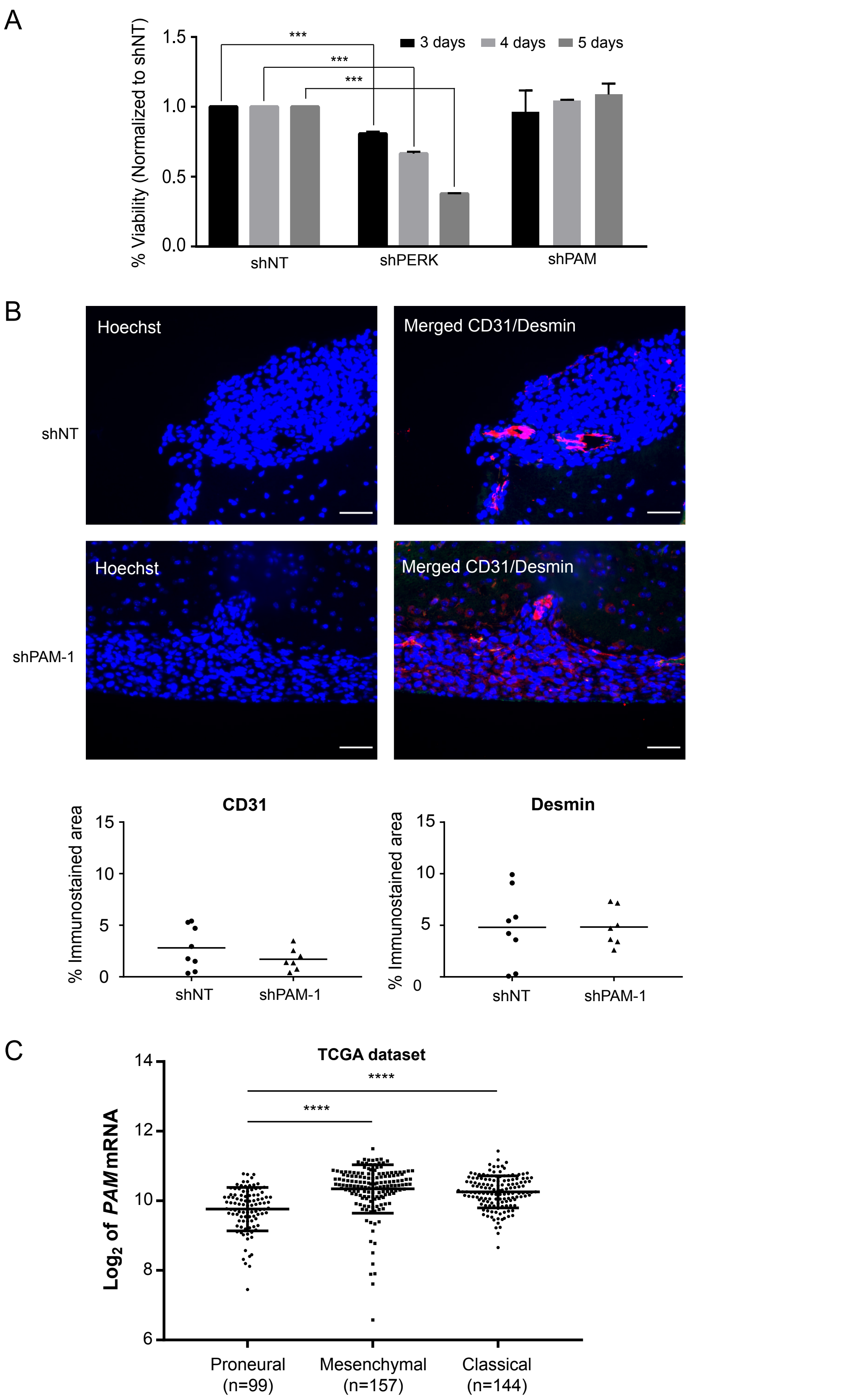

Supplementary Figure S7. A) Cell Titer-Glo® luminescent cell viability assay showing the effect of PERK or PAM on LN229 cell viability determined 3, 4, and 5 days post

virus infection. The values correspond to three biological replicates normalized to the respective shNT control ± SEM (t-test with p-value < 0.05*, <0.01** and < 0.001***). **B)** Representative images of shNT and shPAM-1 tumor sections presented in Fig. 7B stained with Hoechst, along with the merged image (scale bar: 50 µm). The scatter dot plots show the % immunostained area for CD31 (endothelial marker) and Desmin (pericyte marker) in the tumor section from one mouse per cohort, 14 days after implantation of tumor cells. **C)** Scatter dot plot showing the expression levels of *PAM* mRNA in glioblastoma patients classified into different subtypes (data retrieved from https://www.cancer.gov/tcga; error bars depict standard deviation in the expression values (t-test with p-value <0.001***)).
